# Supplementary material for: Synthesis and Properties of 1,3-Disubstituted Ureas Containing (Adamantan-1-yl)(phenyl)methyl Fragment Based on One-Pot Direct Adamantane Moiety Inclusion
Source: Molecules. 2023 Apr 19;28(8):3577. doi: 10.3390/molecules28083577 (PMC10143370; doi:10.3390/molecules28083577)

Synthesis and Properties of 1,3-Disubstituted Ureas Containing (Adamantan-1-yl)(phenyl)methyl Fragment Based on One-Pot Direct Adamantane Moiety Inclusion

Vladimir Dyachenko [1,2], Dmitry Danilov [1], Yaroslav Kuznetsov [1], Semyon Moiseev [2], Vladimir Mokhov [1], Vladimir Burmistrov [1,2]* and Gennady Butov [1,2]

1 Department of Technology of Organic and Petrochemical Synthesis, Volgograd State Technical University (VSTU), 28 Lenin Avenue, Volgograd 400005, Russia; gmbutov@mail.ru (G.B.)

2 Volzhsky Polytechnic Institute (Branch), Volgograd State Technical University (VSTU), 42a Engels Street, Volzhsky 404121, Russia

* Correspondence: vburmistrov@vstu.ru

Supplementary materials

^{1}H NMR, ^{13}C NMR, ^{19}F NMR and Mass spectra

Figure S1. Chromatogram of compound 3b

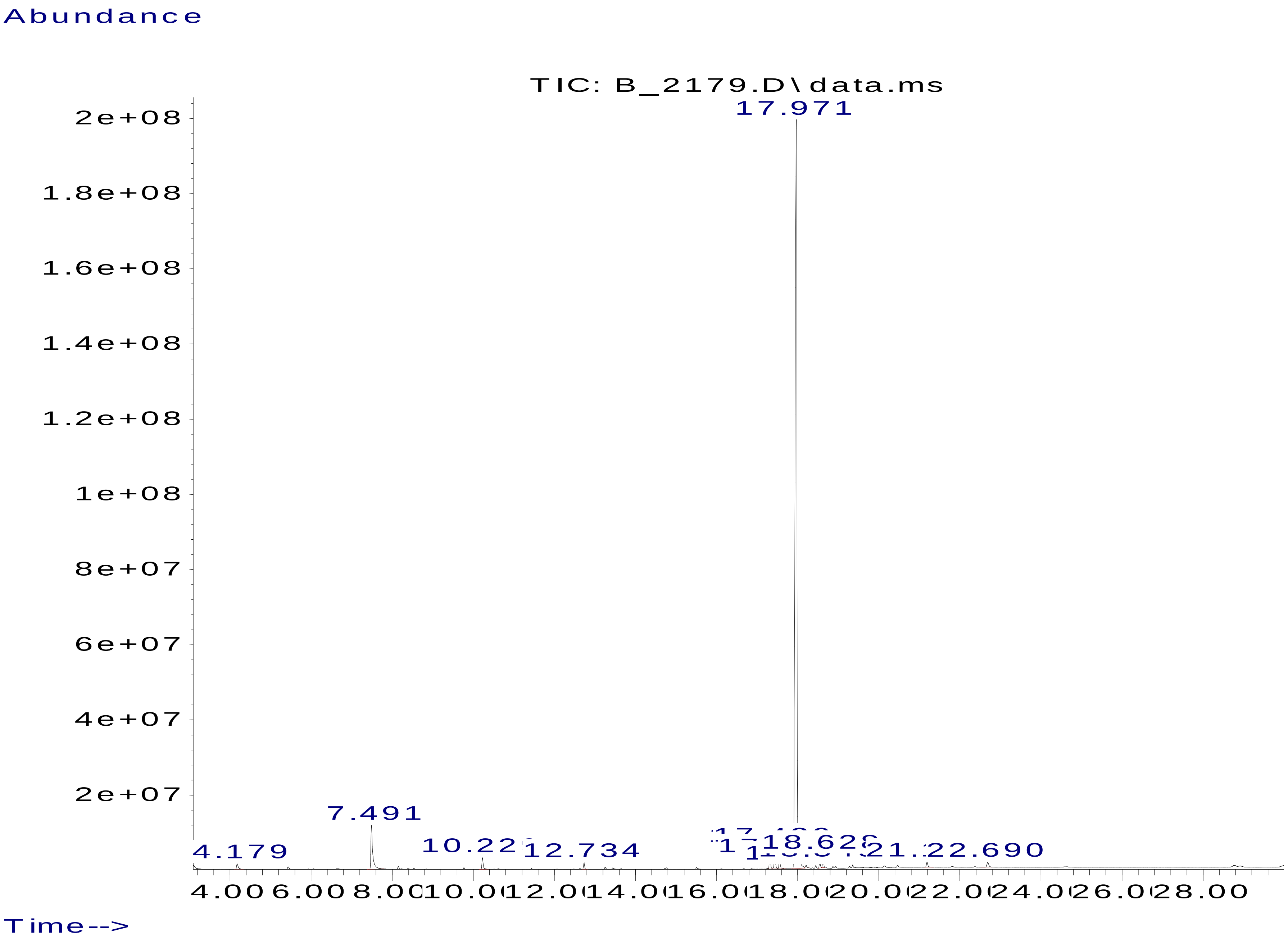

Figure S2. Mass spectrum of compound 3b

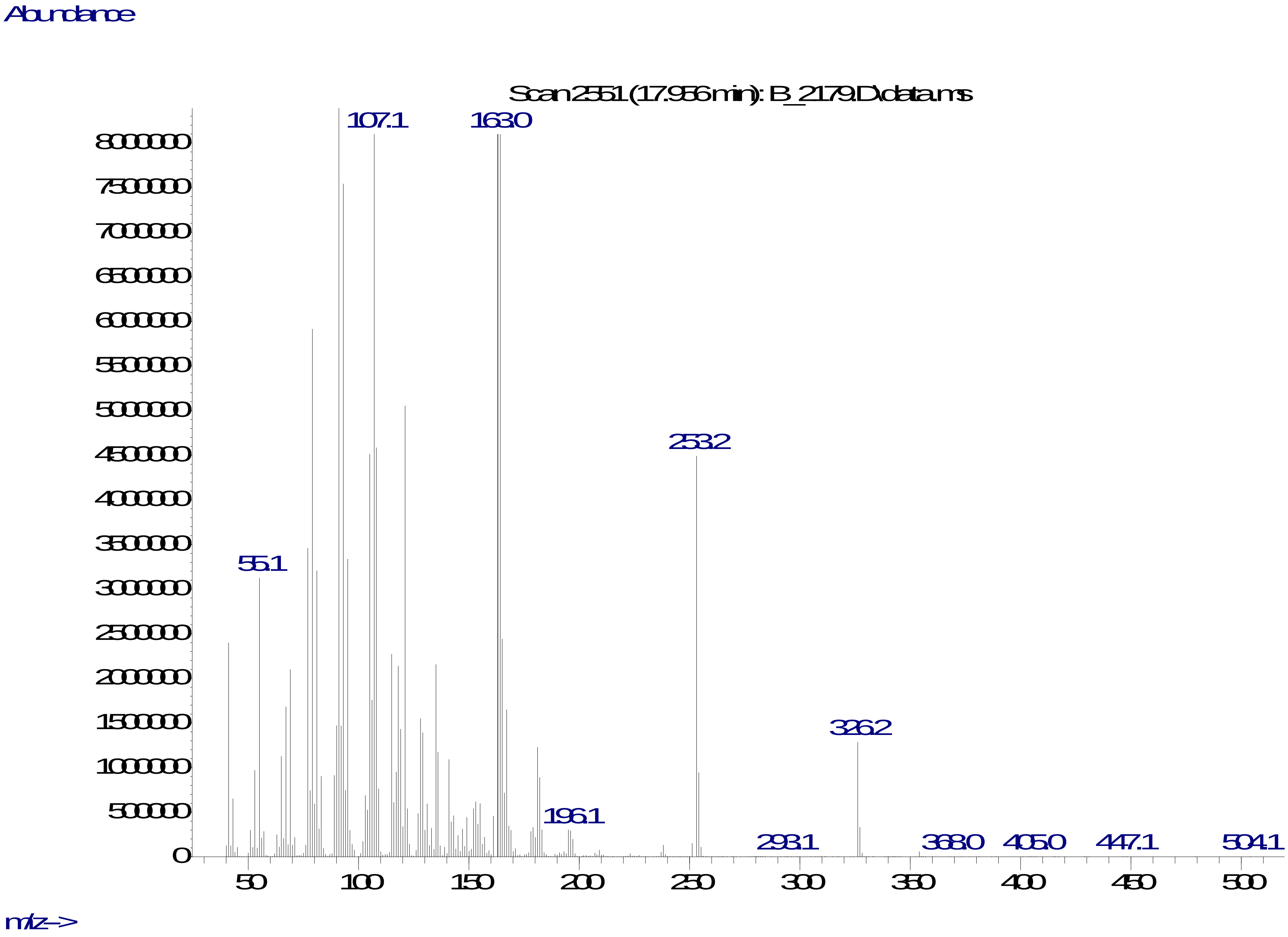

Figure S3. Chromatogram of compound 4b

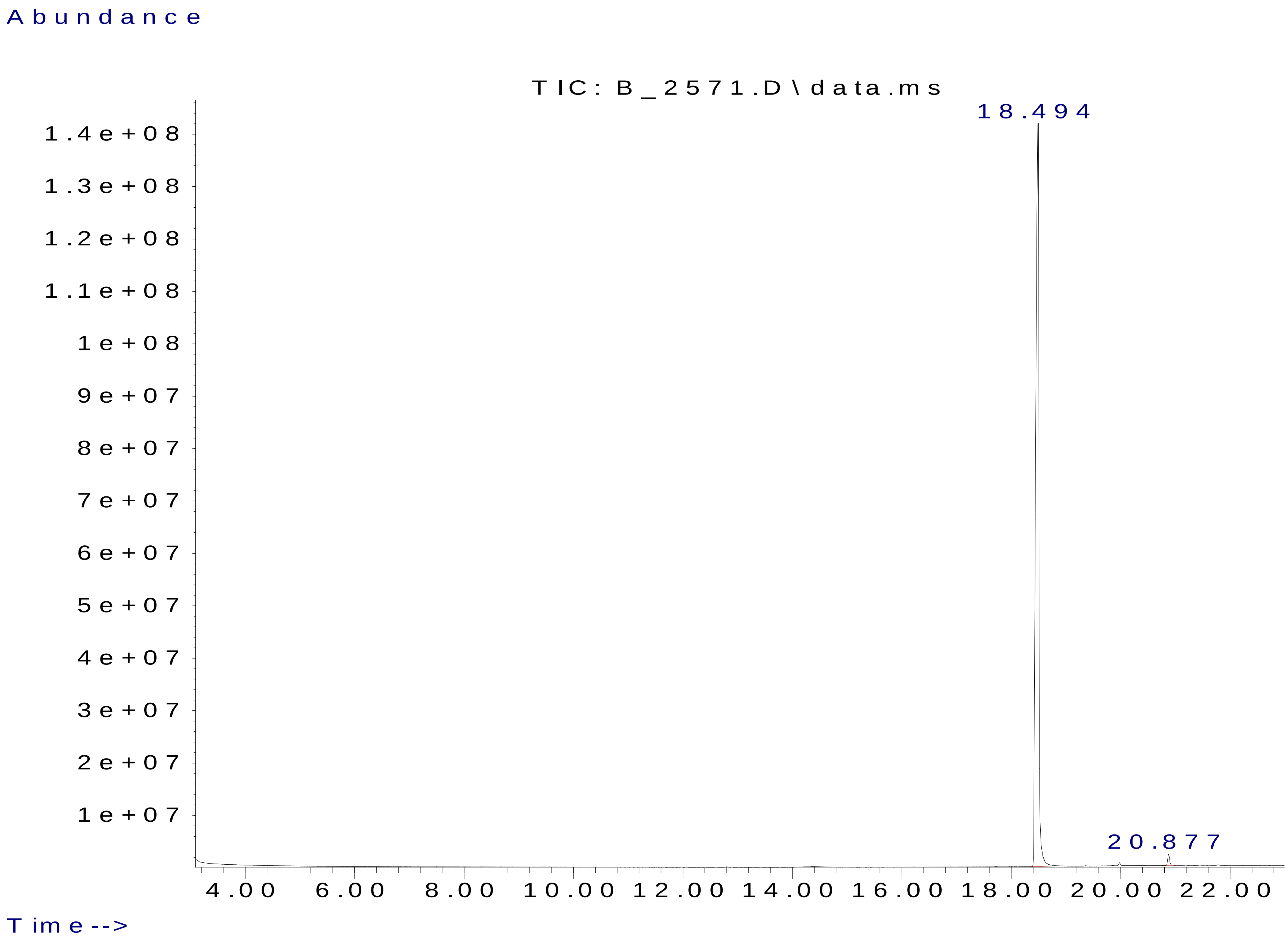

Figure S4. Mass spectrum of compound 4b

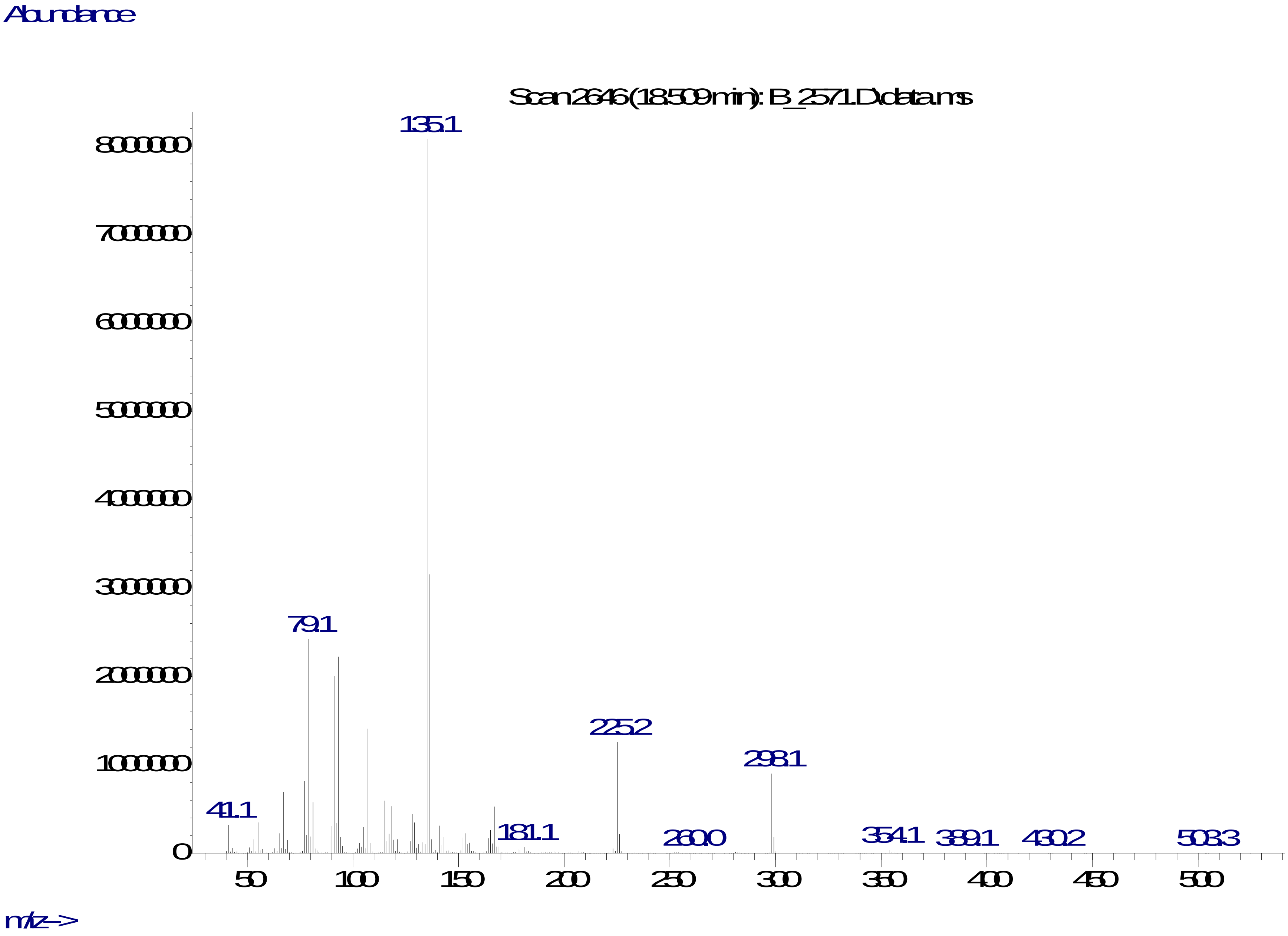

Figure S5. Chromatogram of compound 5b

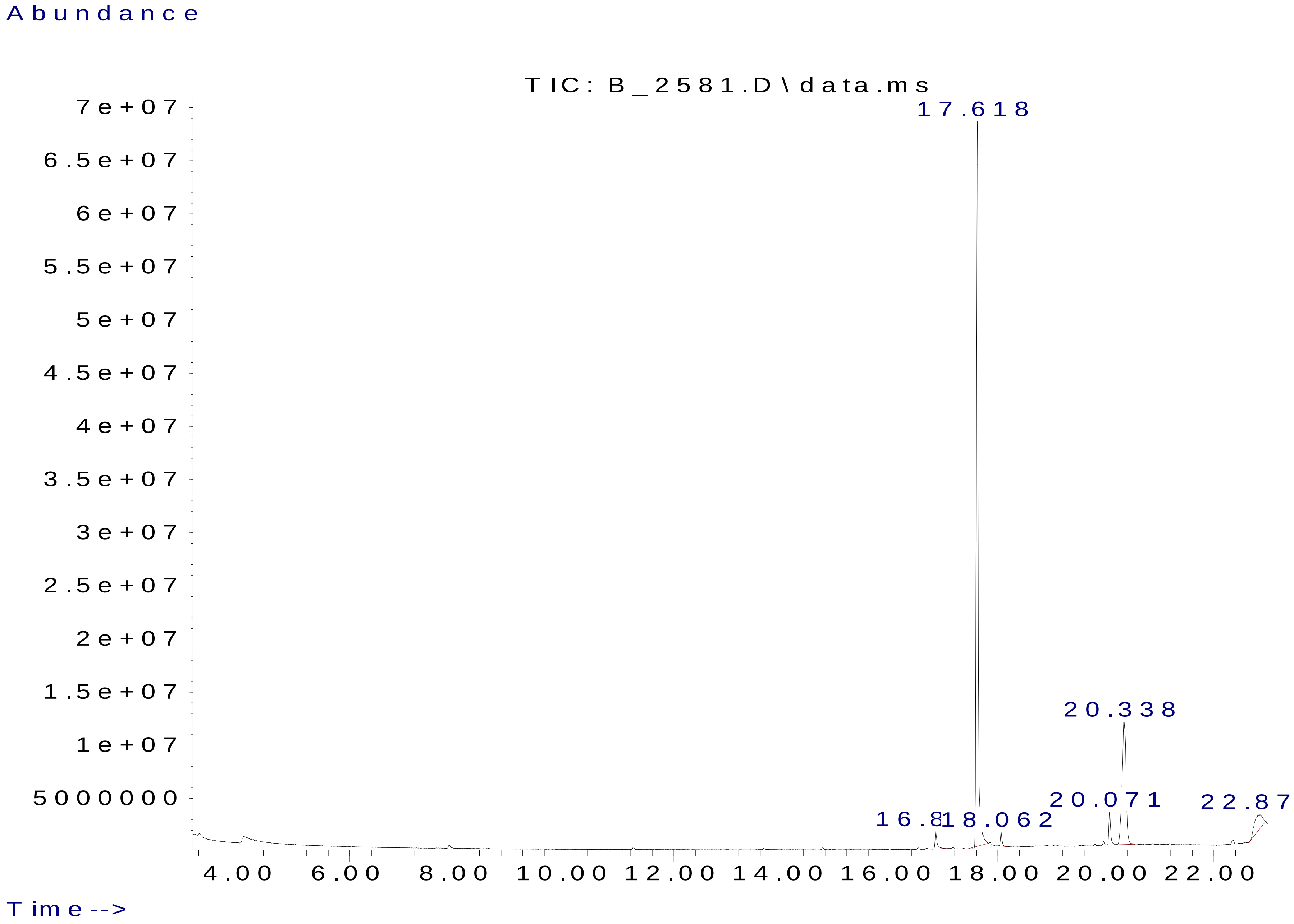

Figure S6. Mass spectrum of compound 5b

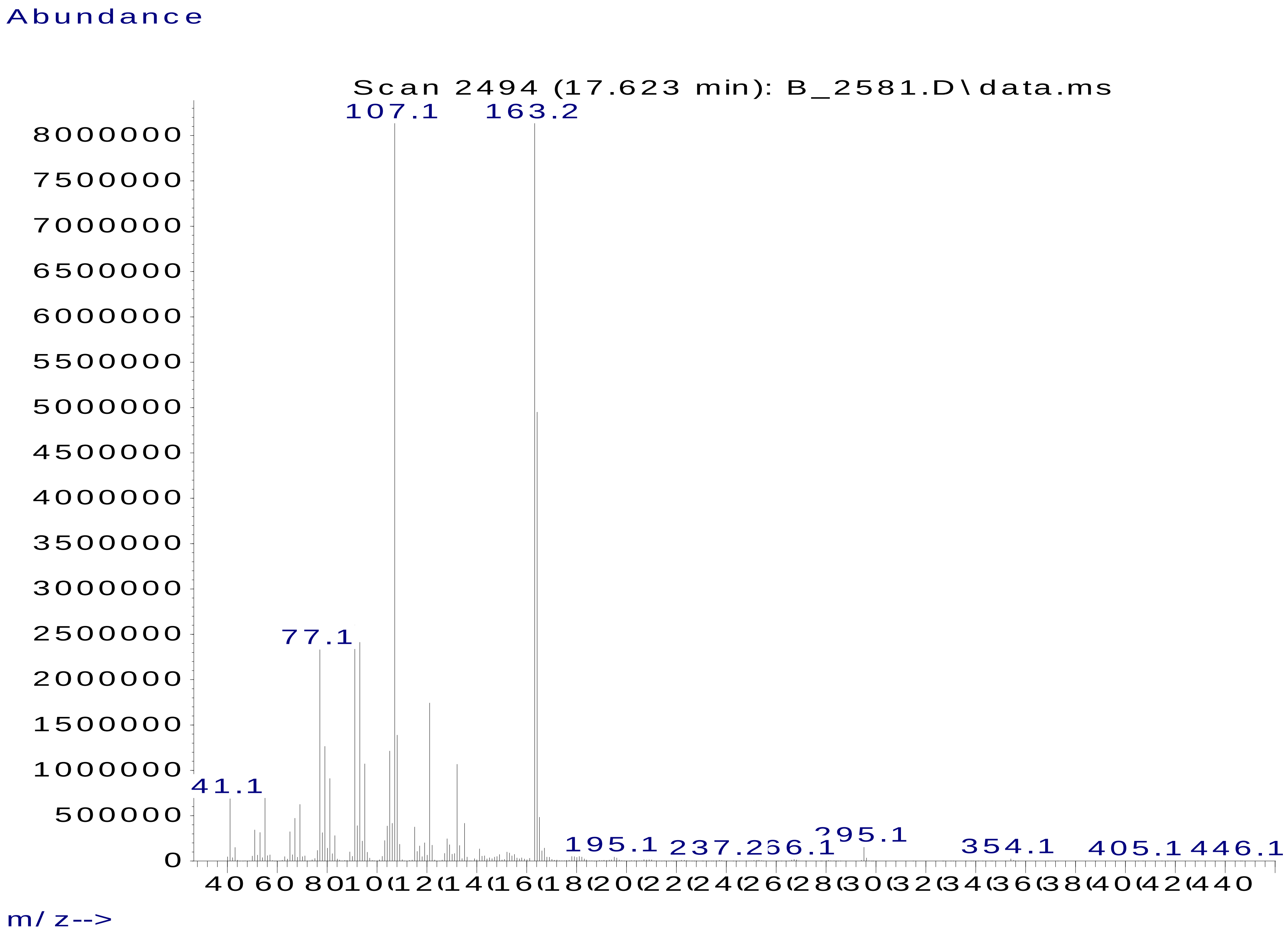

Figure S7. Chromatogram of compound 6a

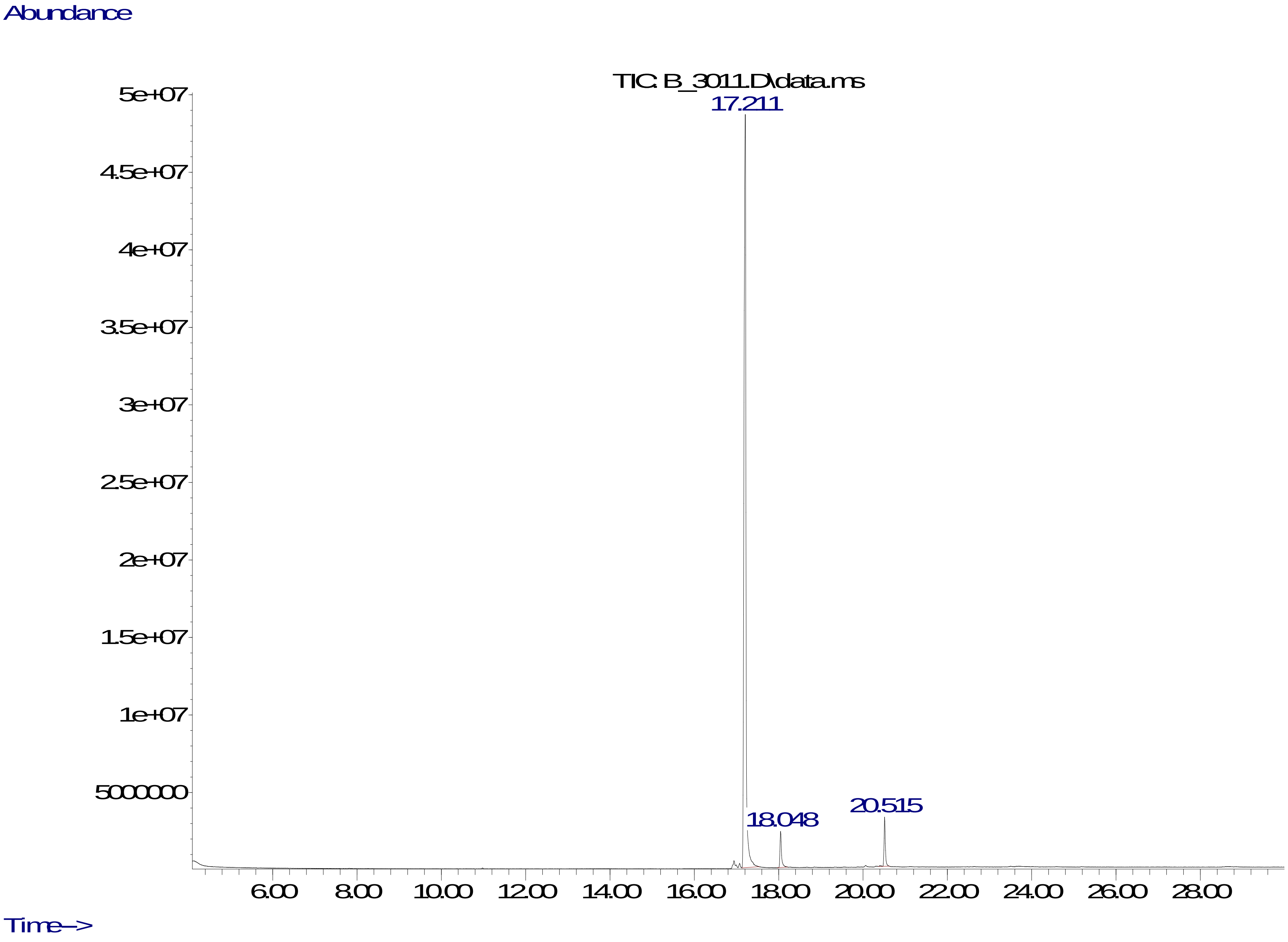

Figure S8. Mass spectrum of compound 6a

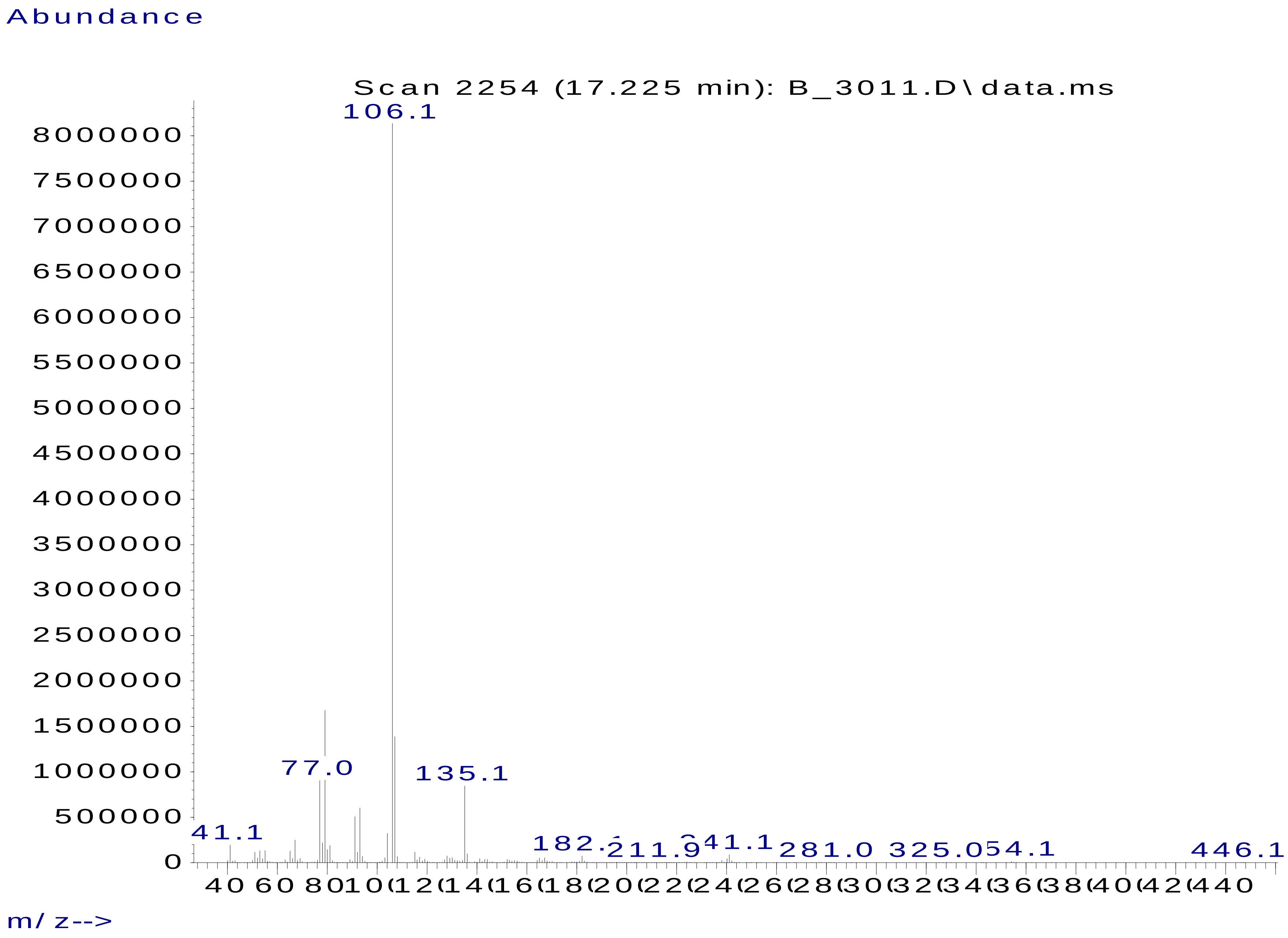

Figure S9. ¹H NMR of compound 6a

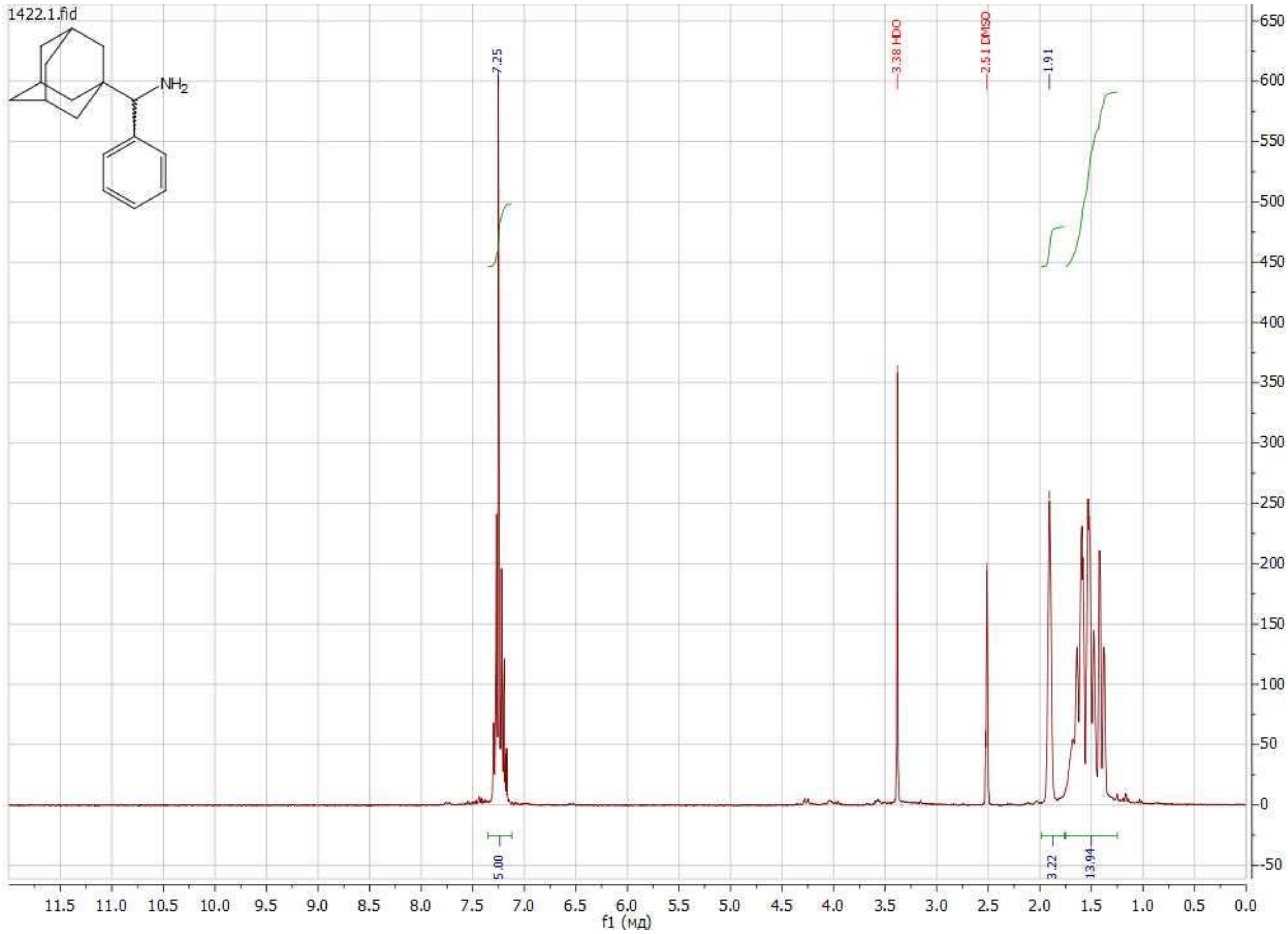

Figure S10. ^{1}H NMR of compound 8a

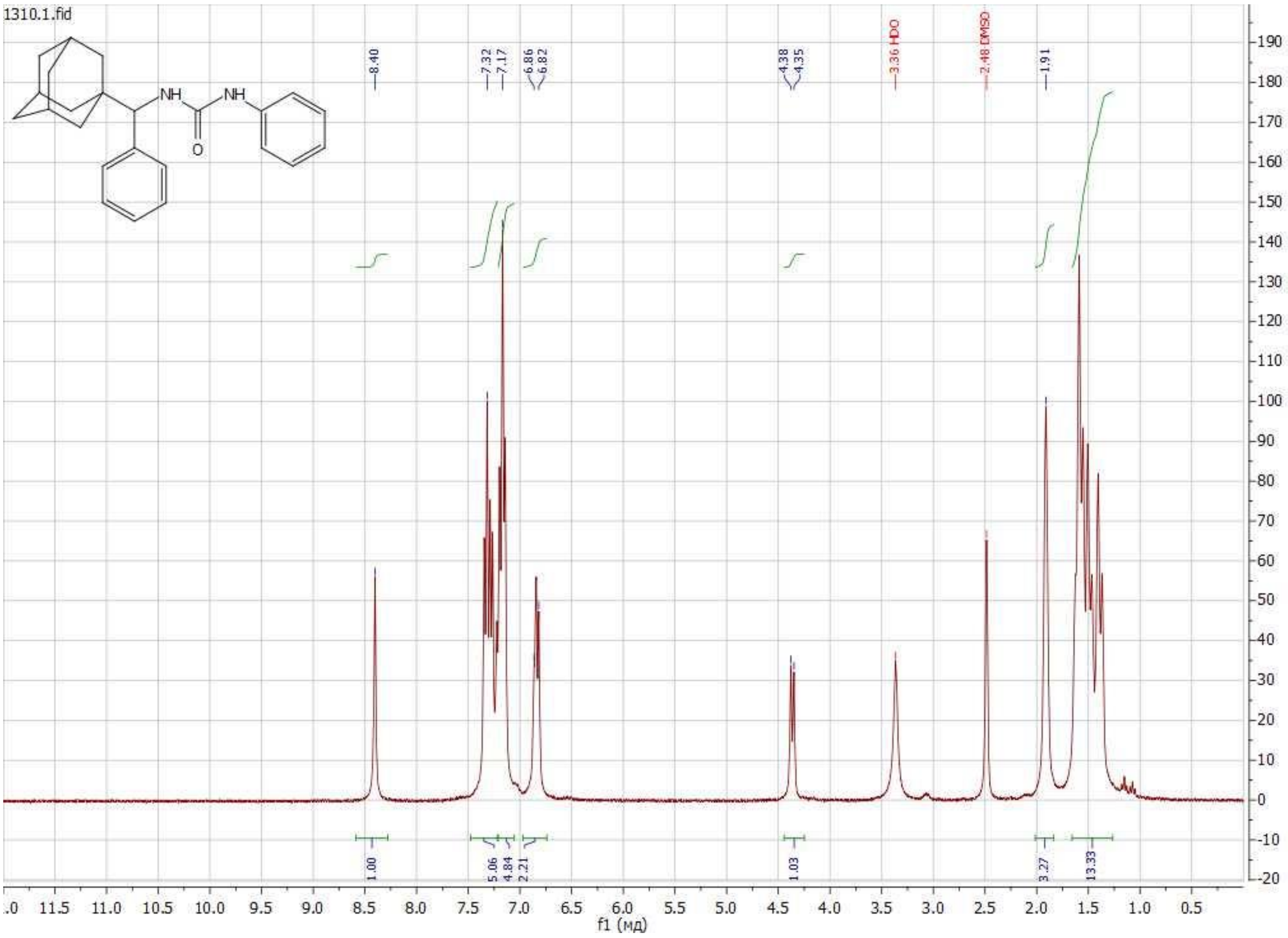

Figure S11. ^{13}C NMR of compound 8a

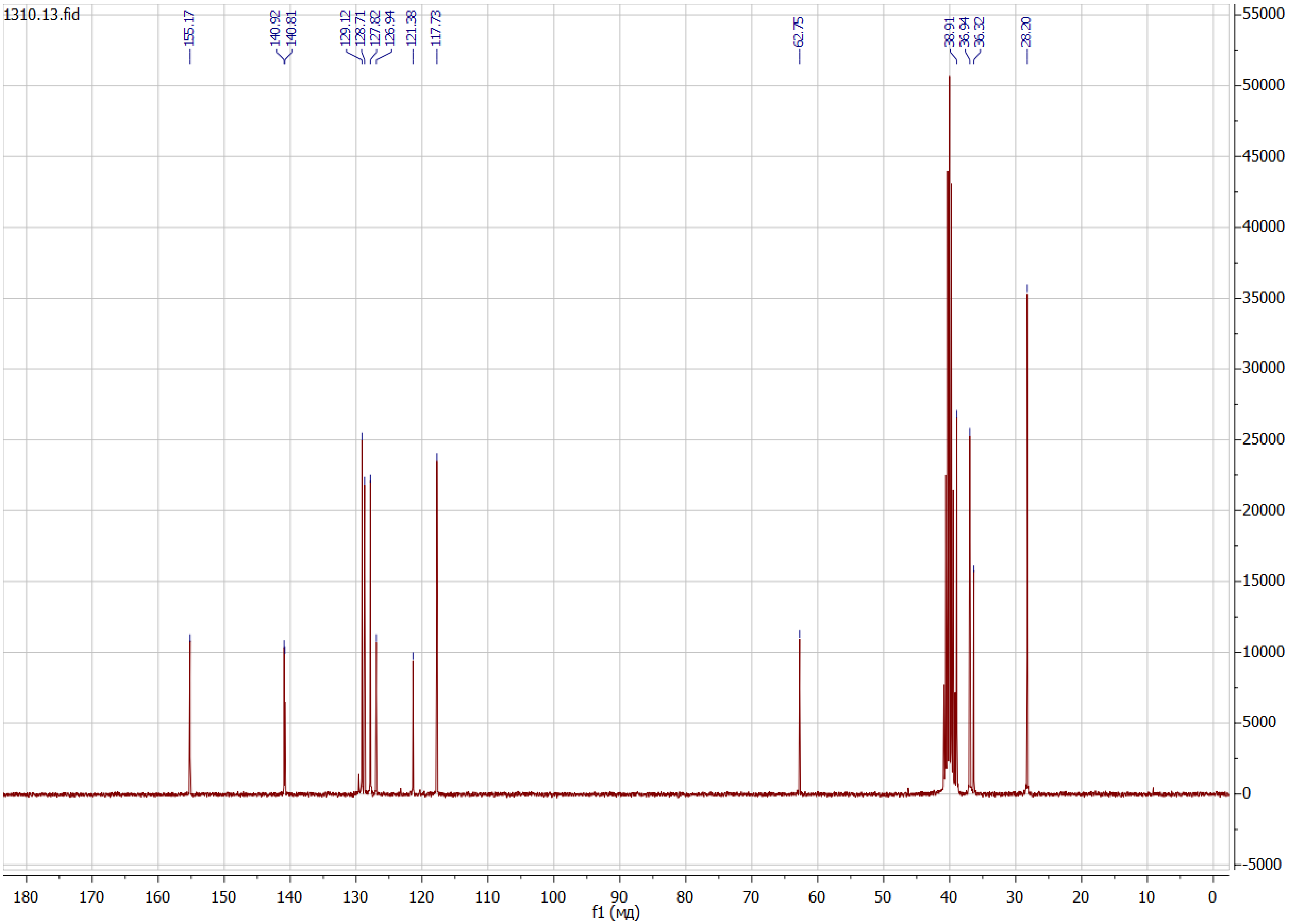

Figure S12. ¹H NMR of compound 8b

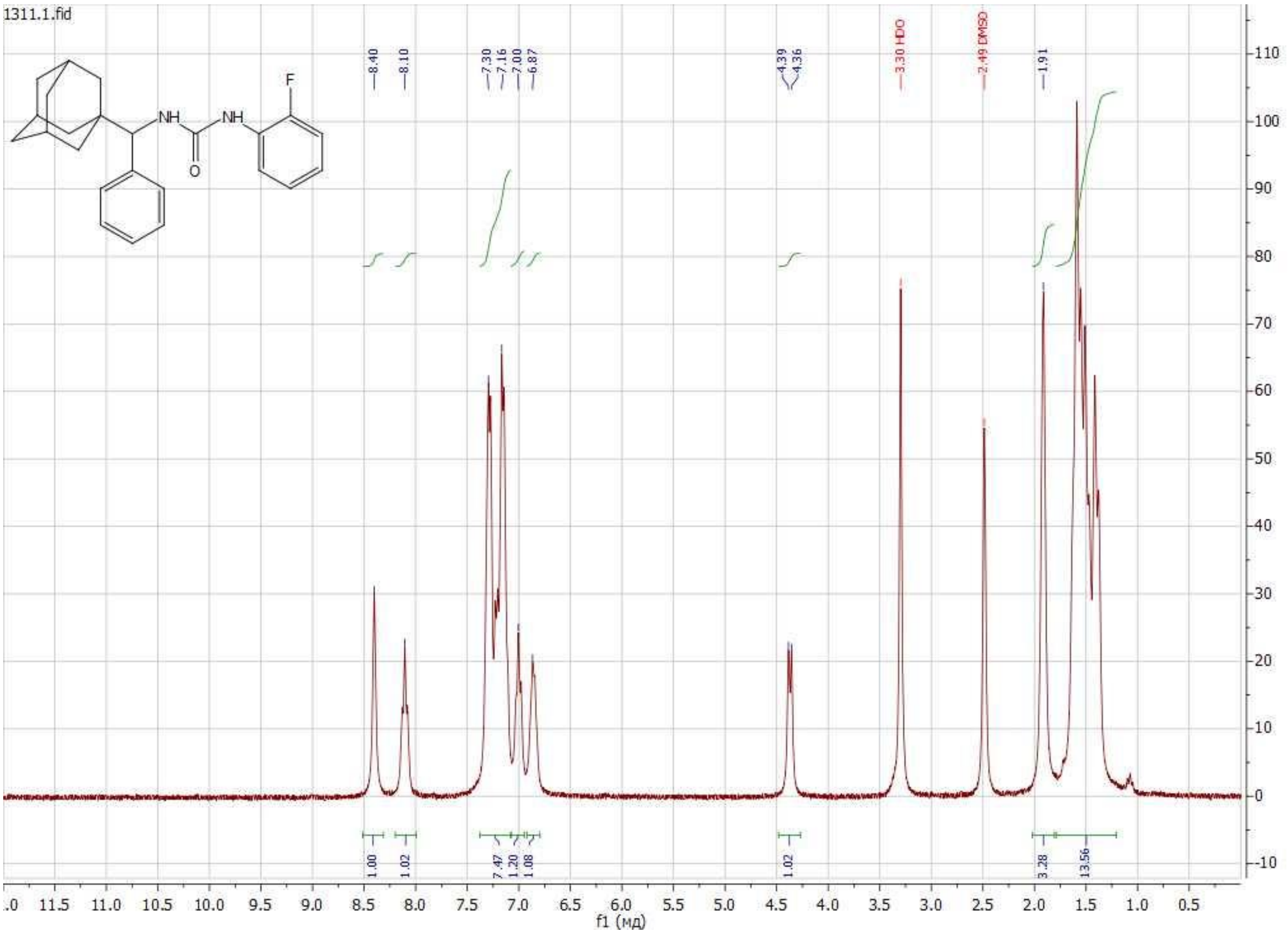

Figure S13. ^{13}C NMR of compound 8b

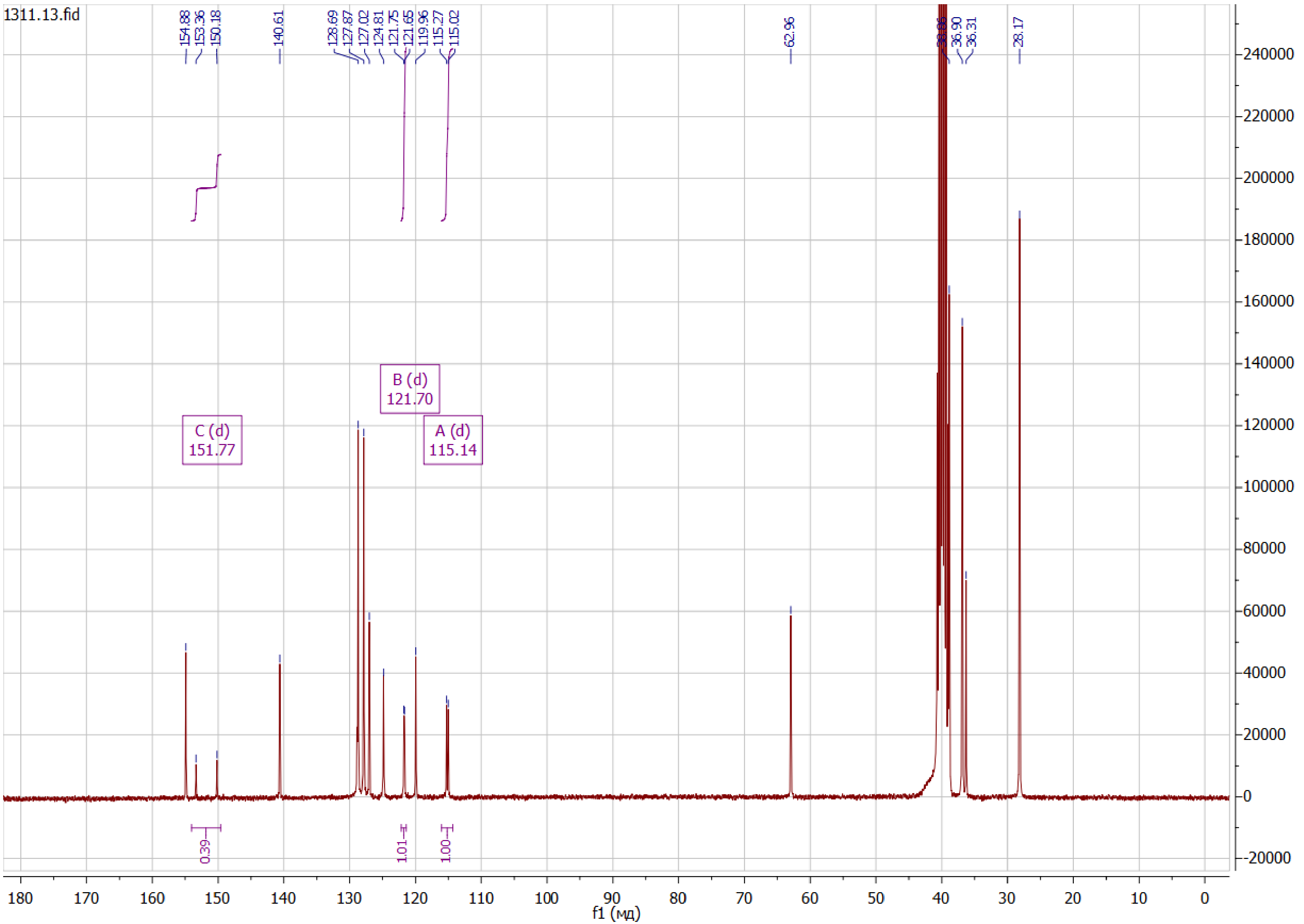

Figure S14. ^{19}F NMR of compound 8b

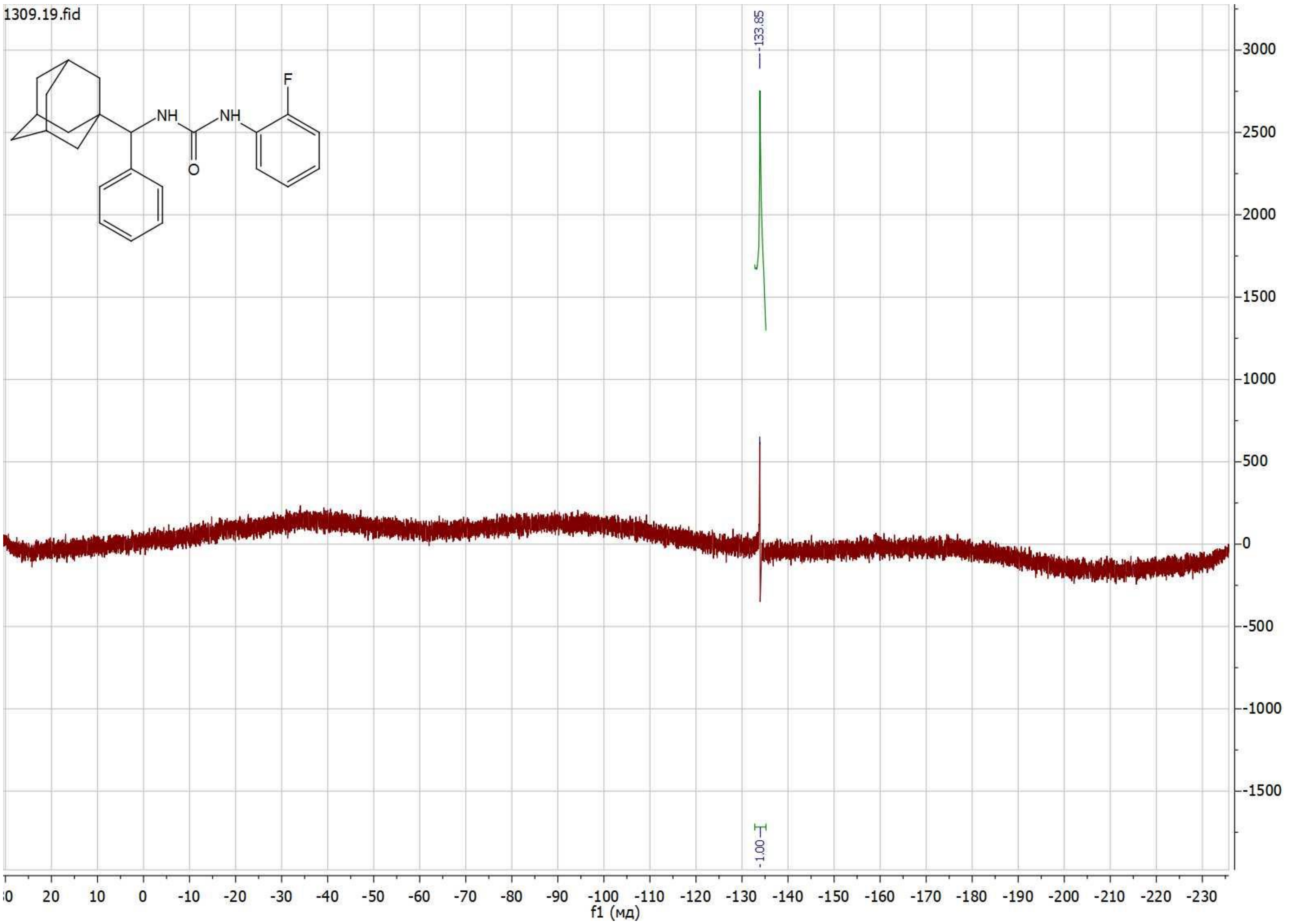

Figure S15. ^{1}H NMR of compound 8c

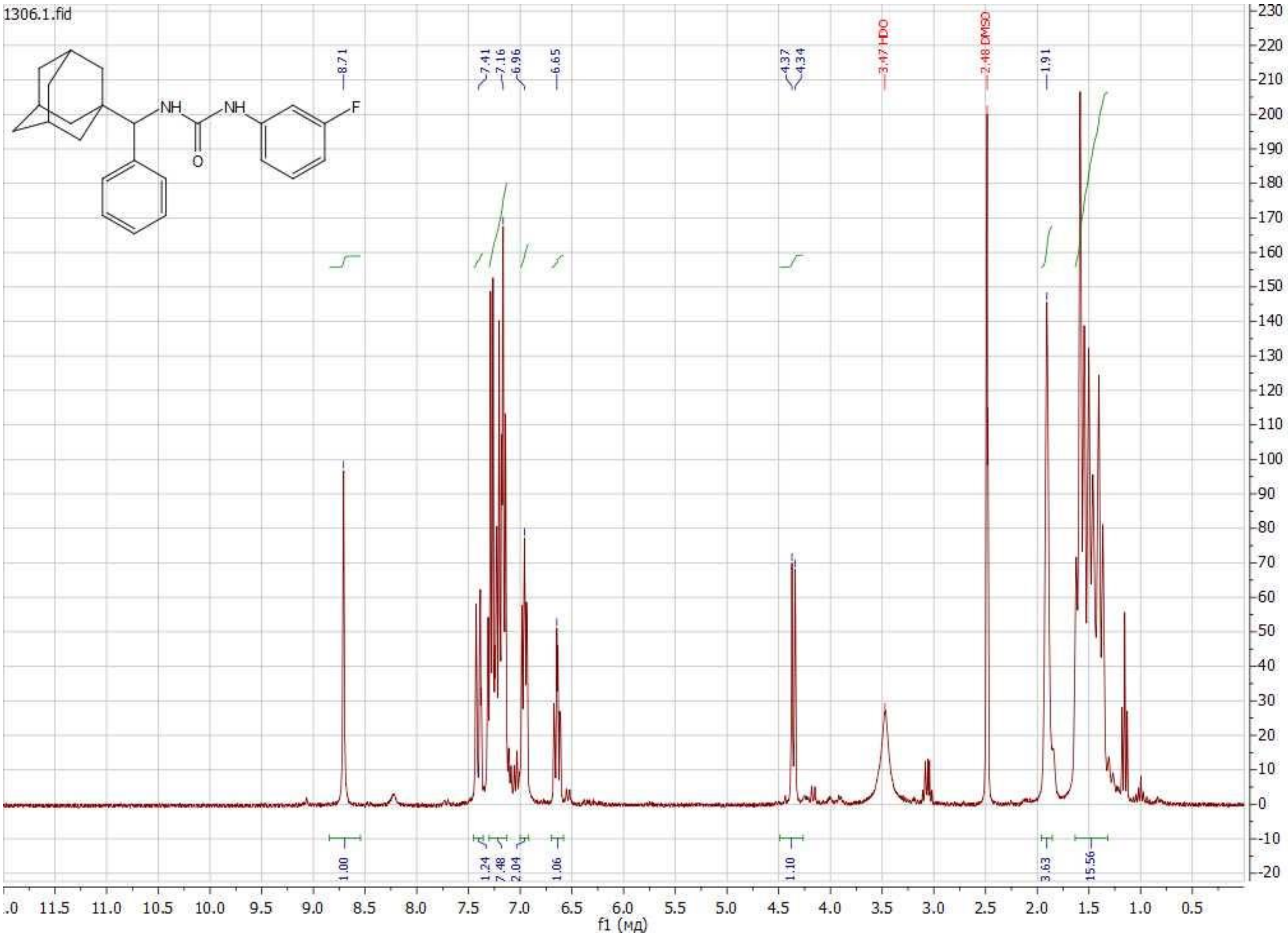

Figure S16. ^{13}C NMR of compound 8c

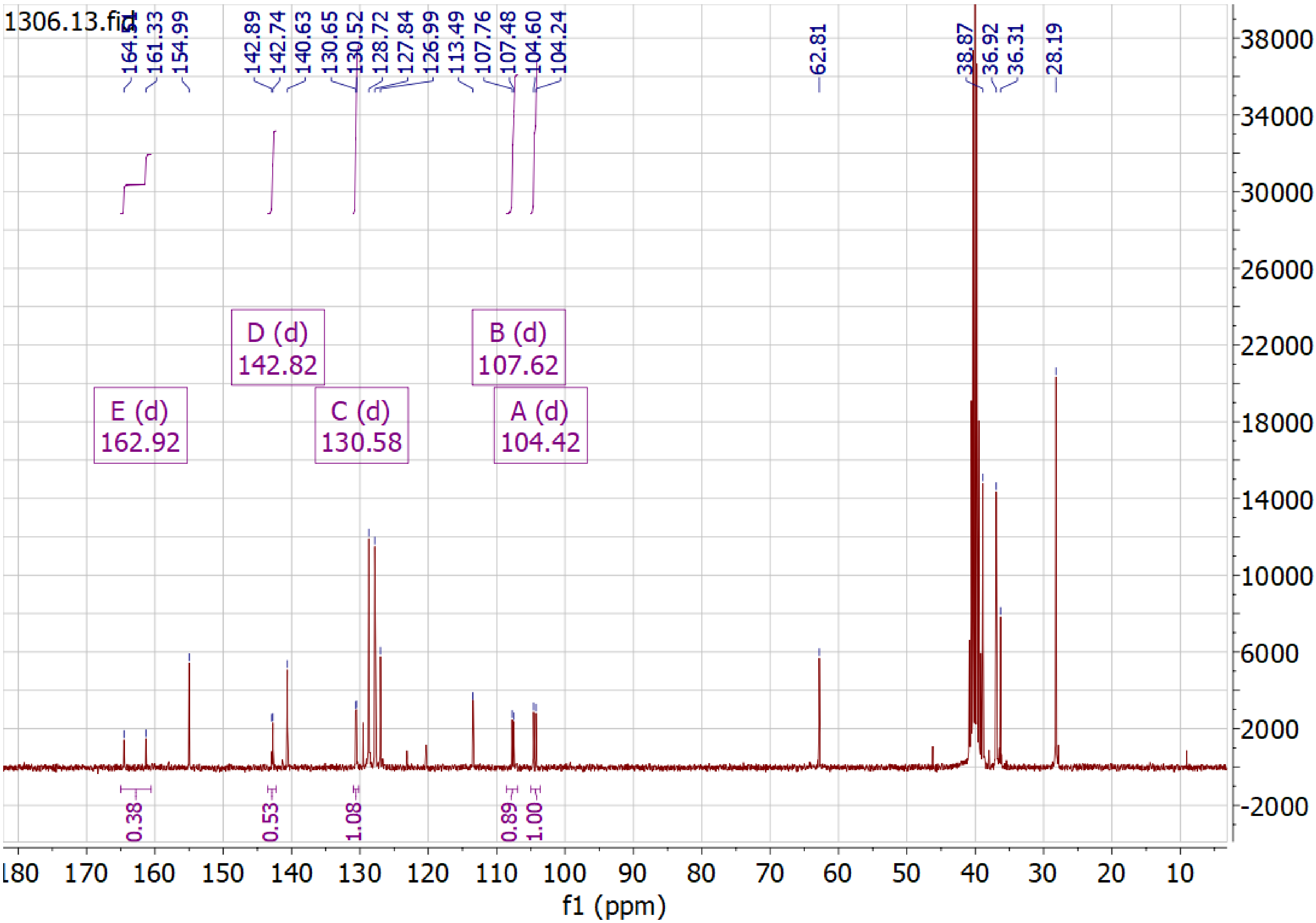

Figure S17. ^{19}F NMR of compound 8c

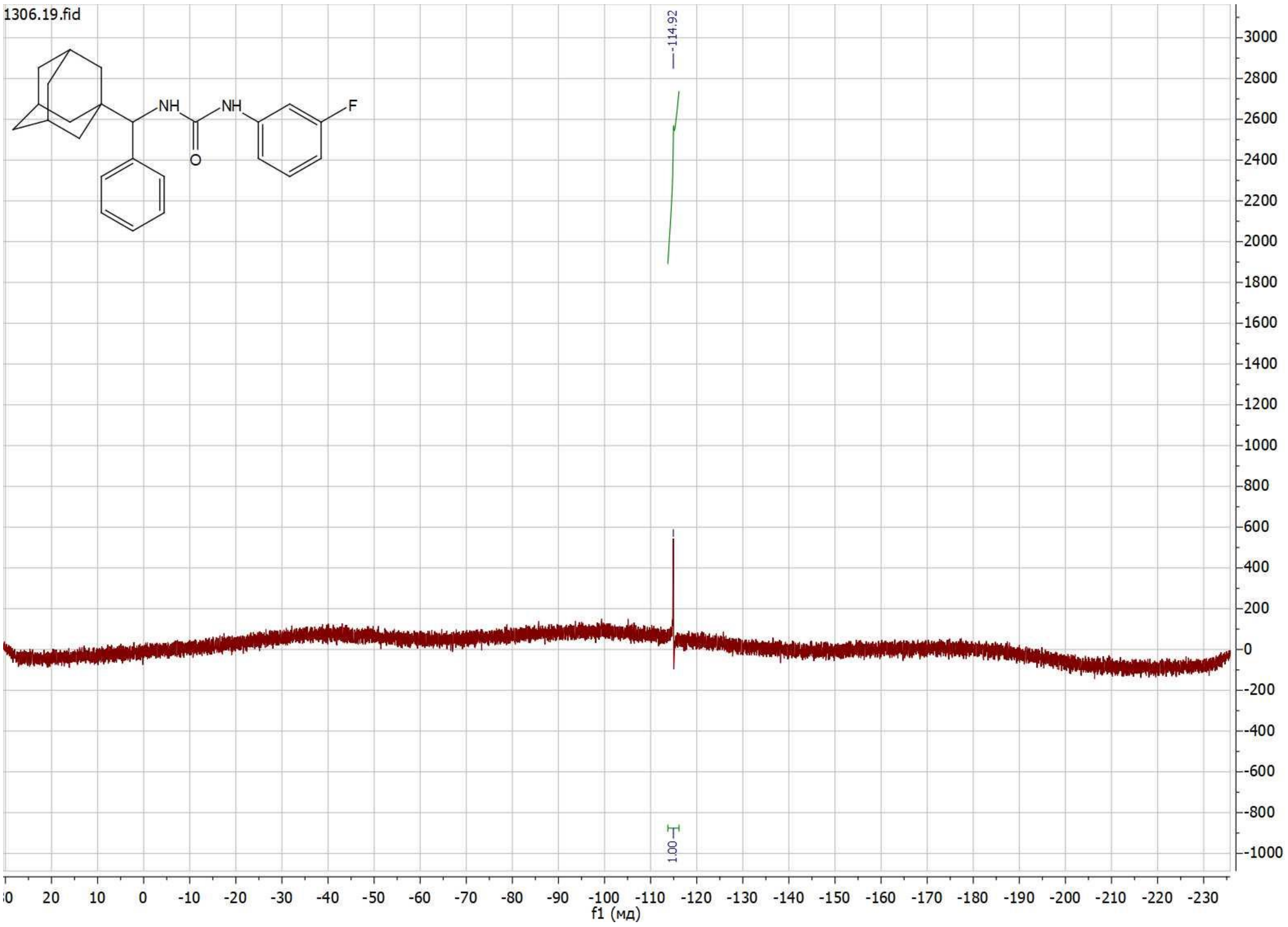

Figure S18. ^{1}H NMR of compound 8d

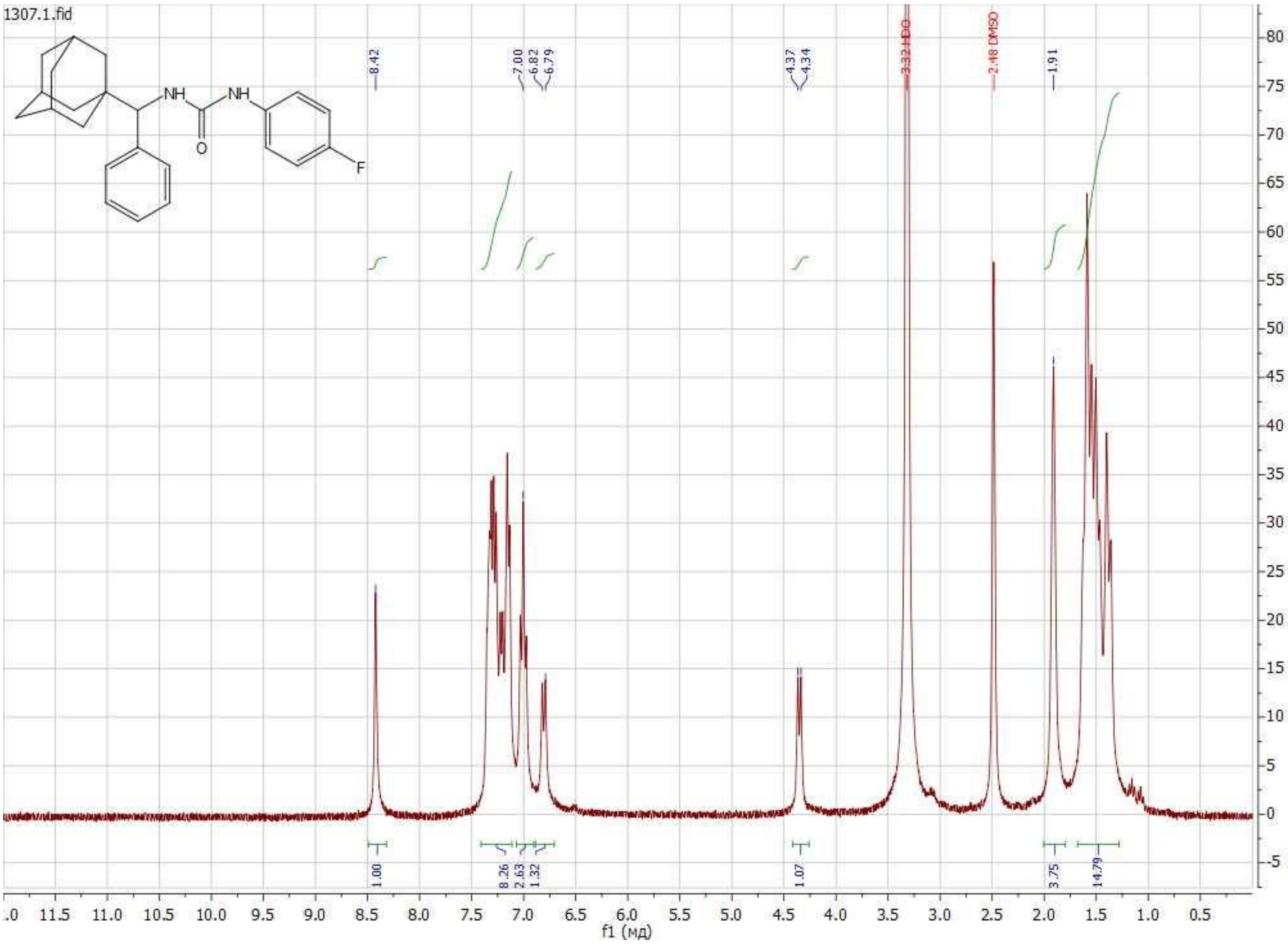

Figure S19. ^{13}C NMR of compound **8d**

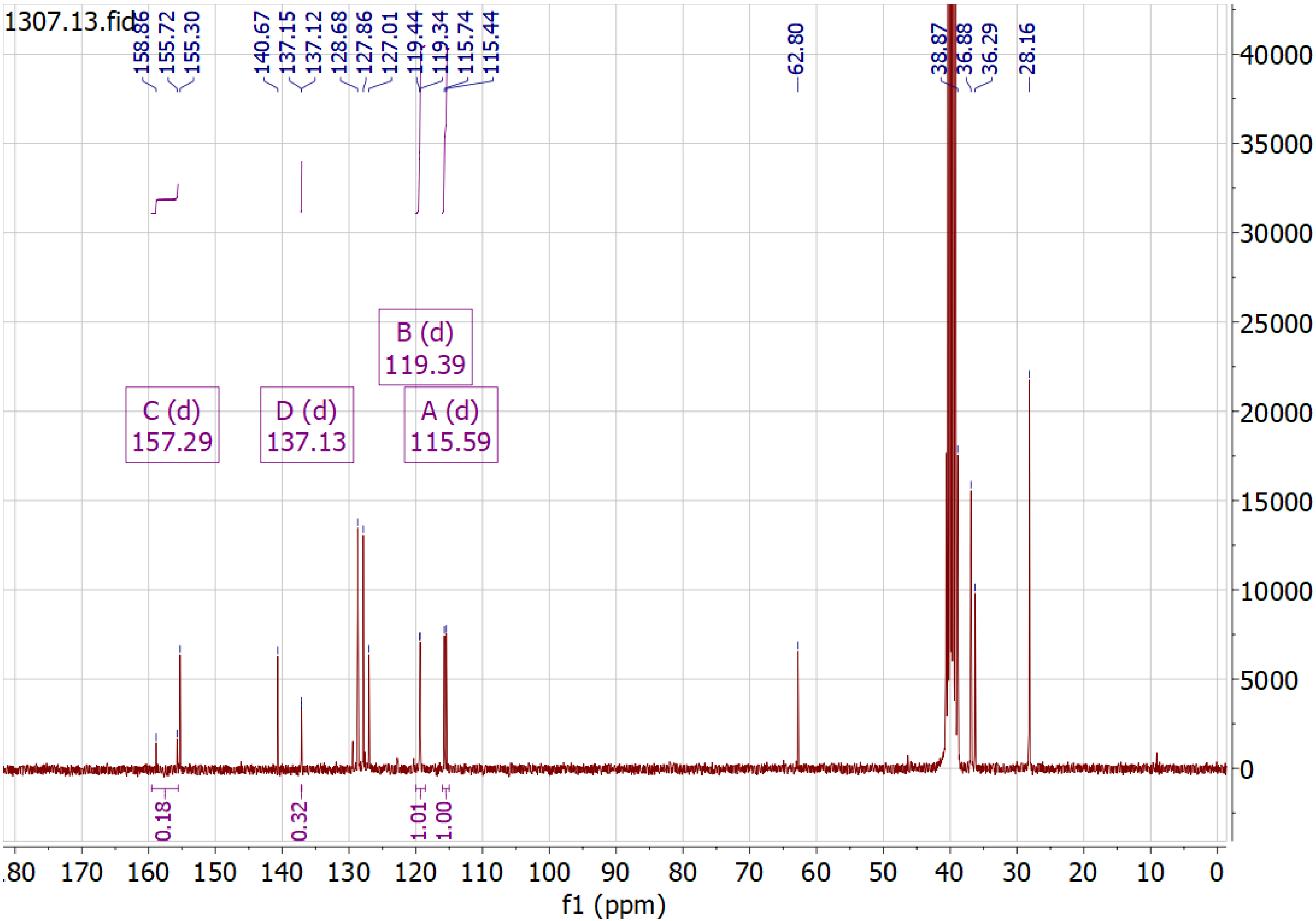

Figure S20. ^{1}H NMR of compound 8e

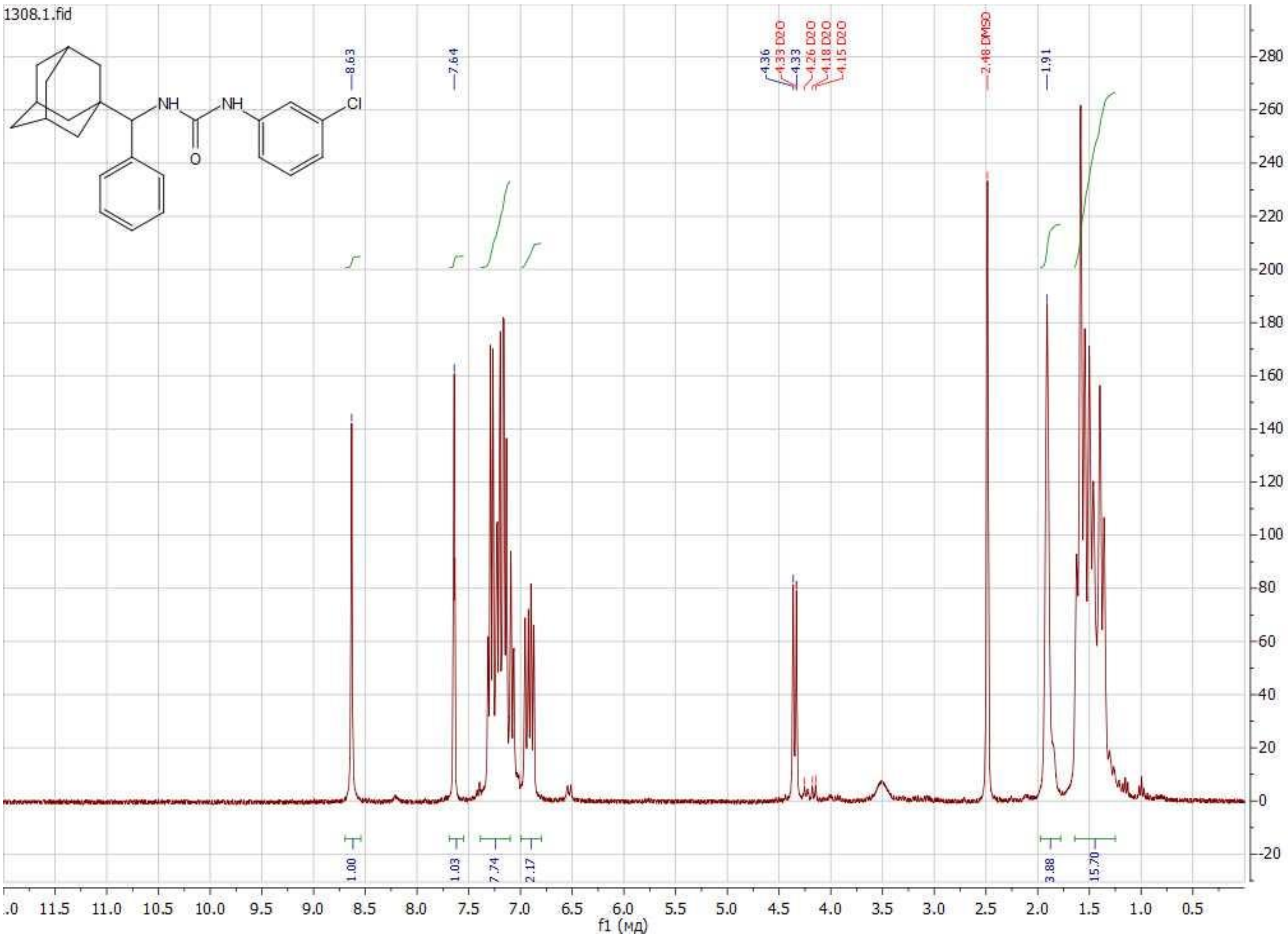

Figure S21 ^{13}C NMR of compound 8e

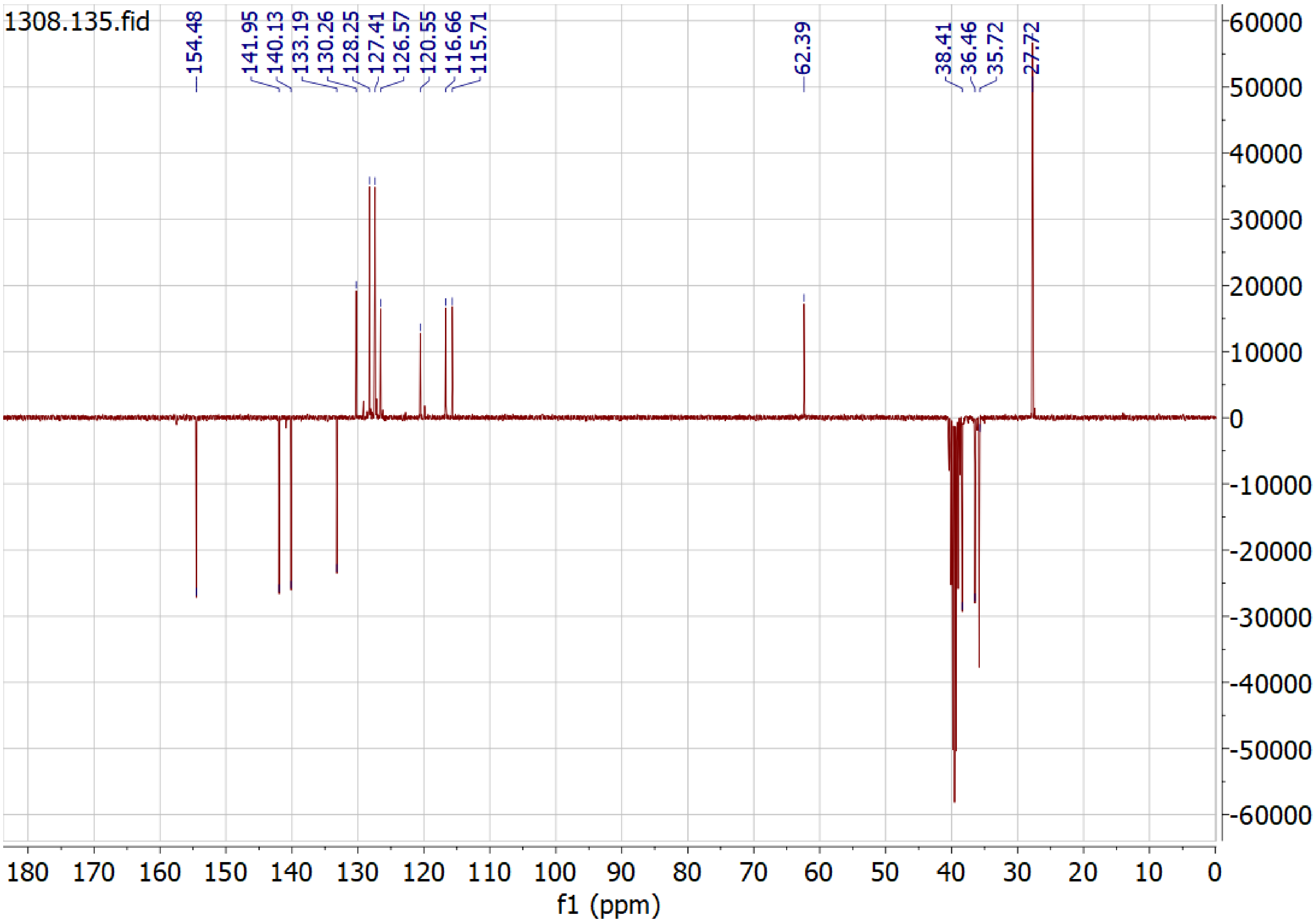

Figure S22. ^{1}H NMR of compound 8f

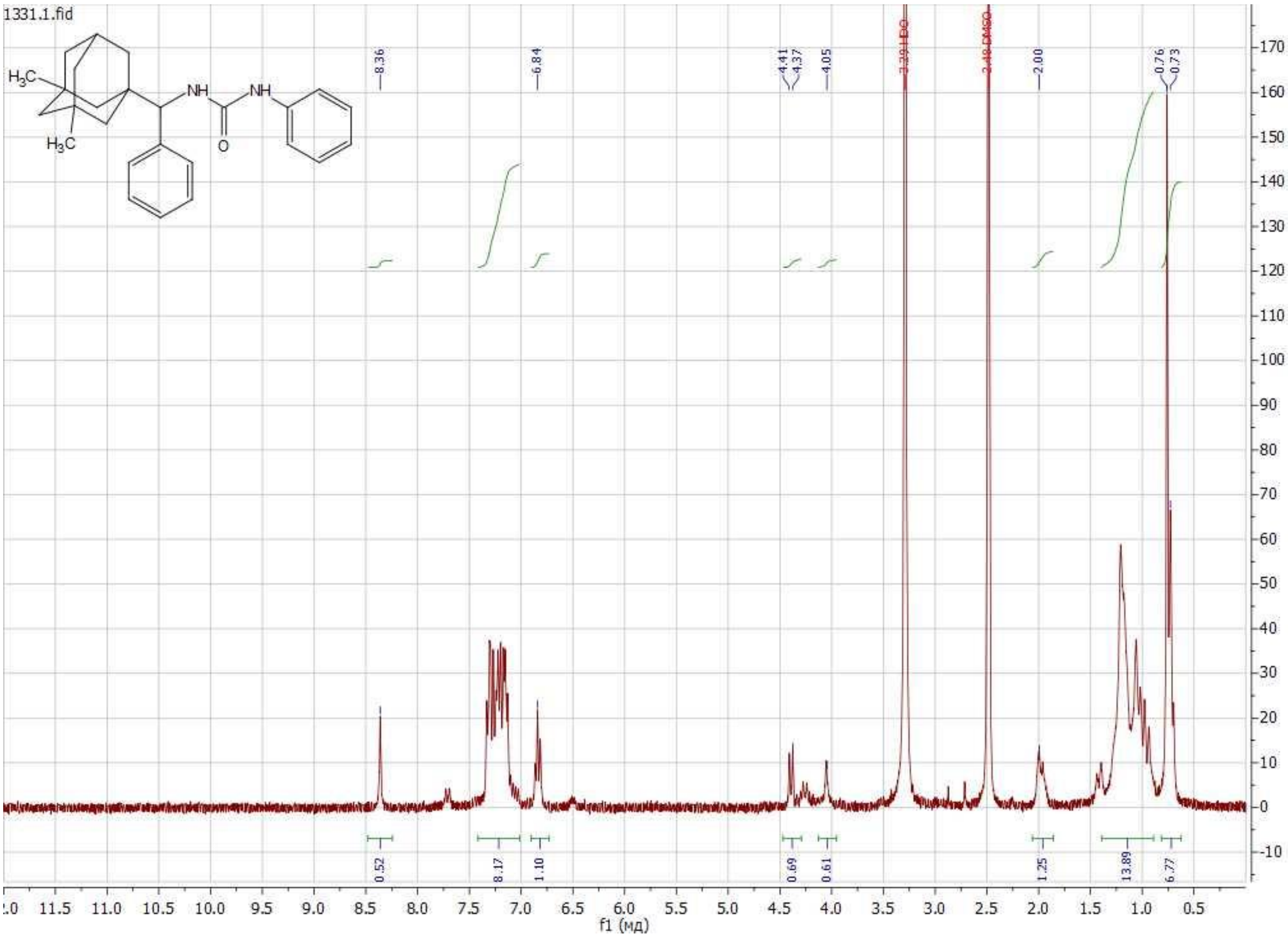

Figure S23. ^{13}C NMR of compound 8f

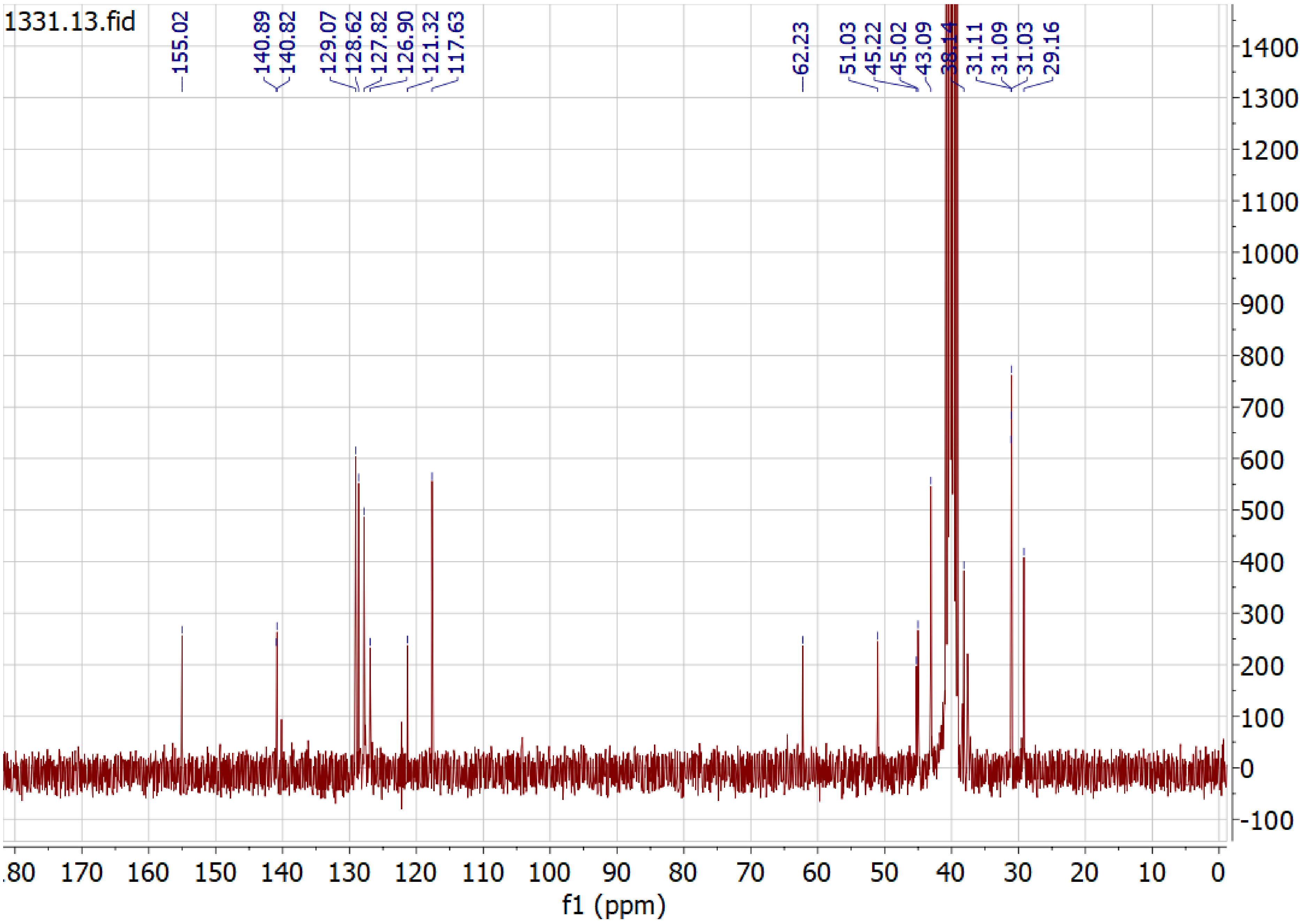

Figure S24. ^{1}H NMR of compound 8g

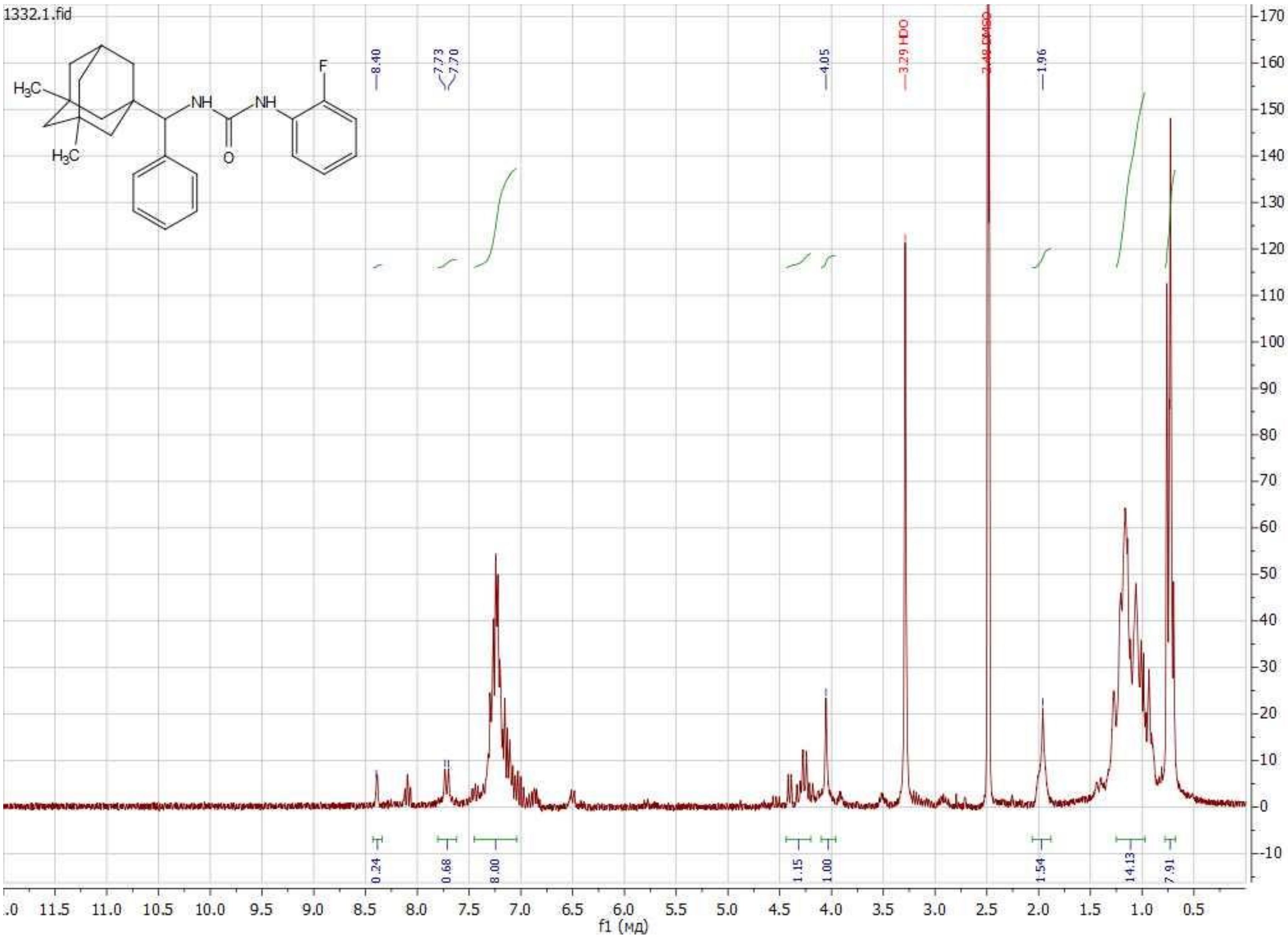

Figure S25. ^{13}C NMR of compound 8g**

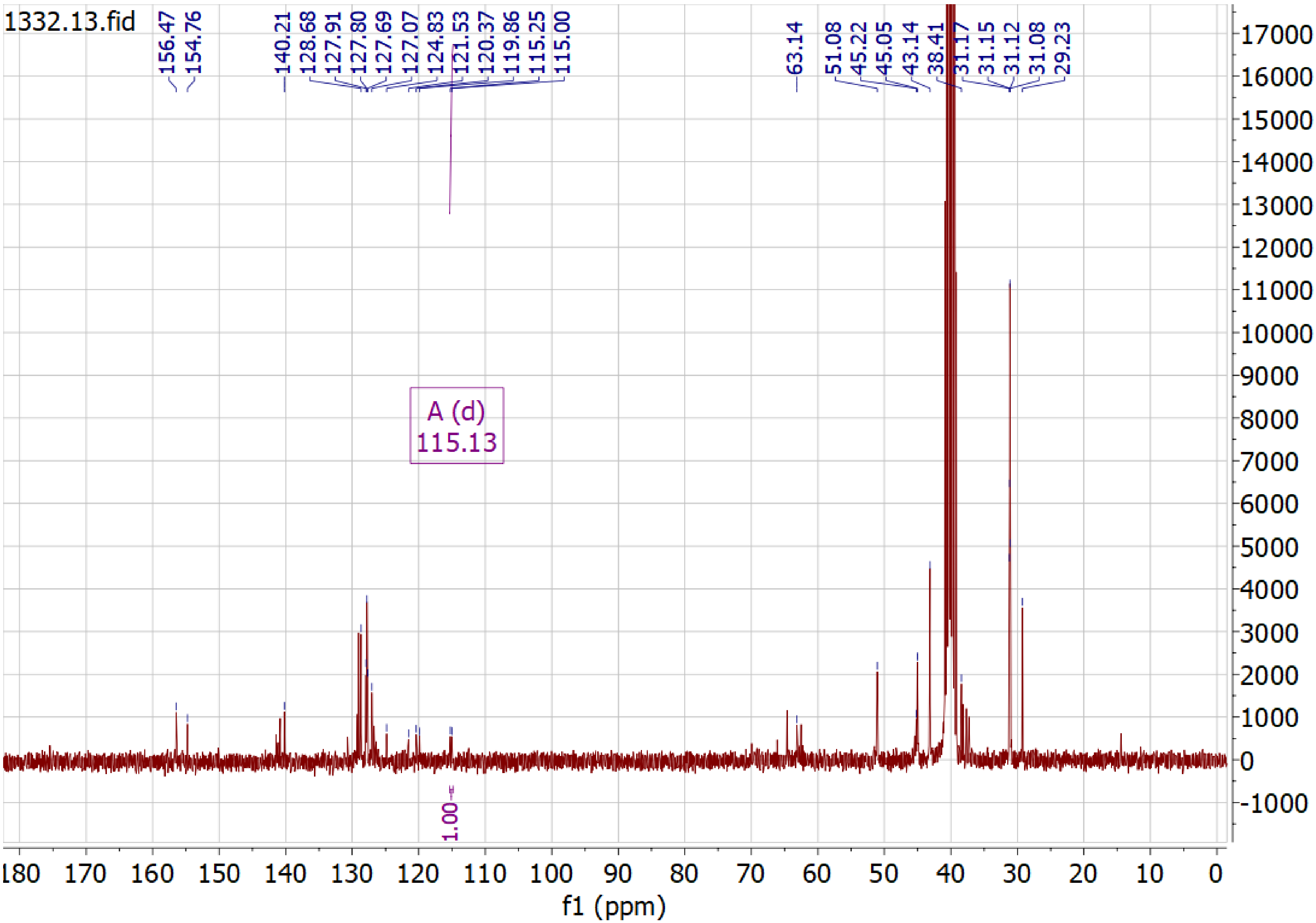

Figure S26. ^{1}H NMR of compound 8h

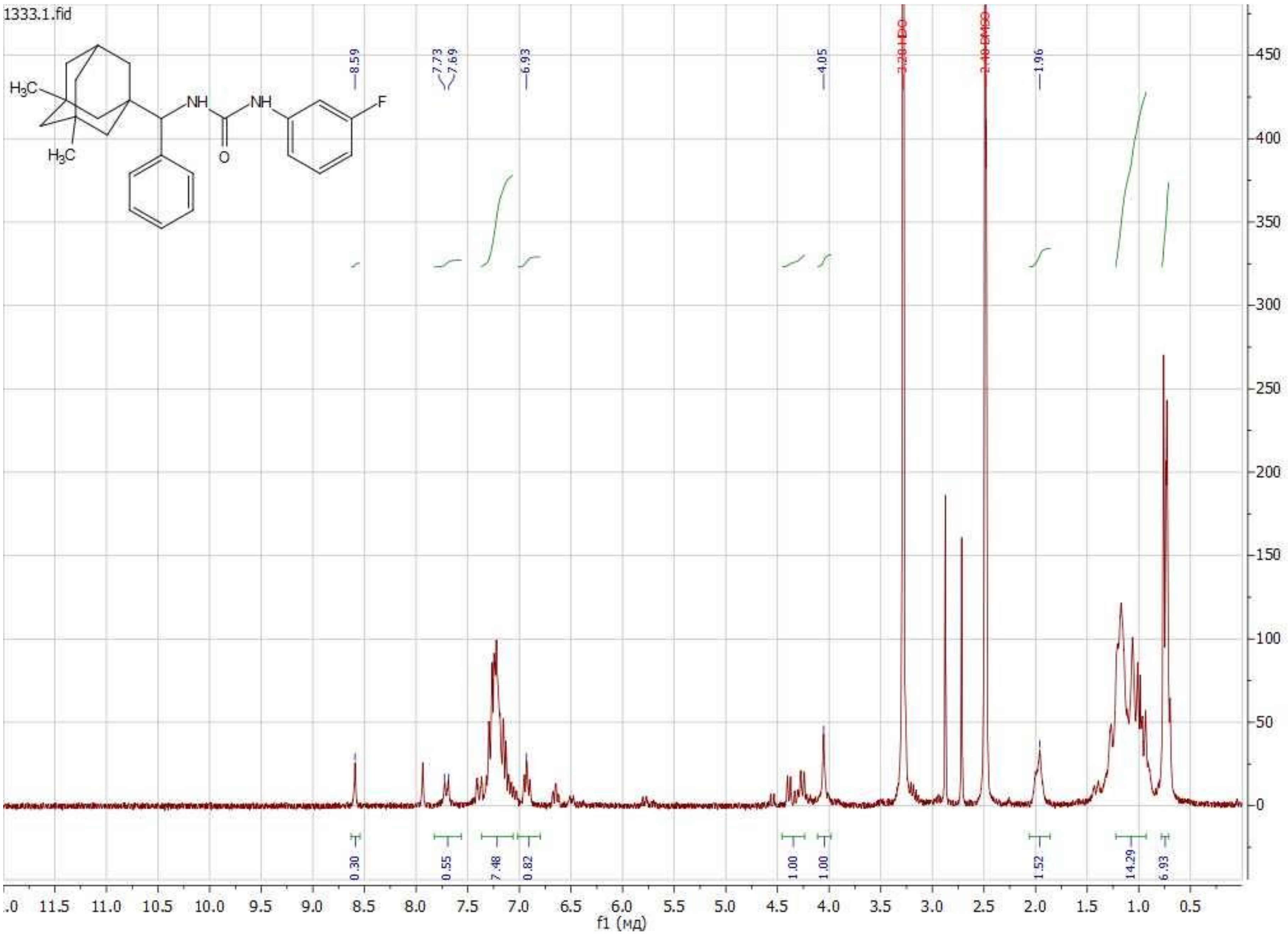

Figure S27. ^{13}C NMR of compound 8h

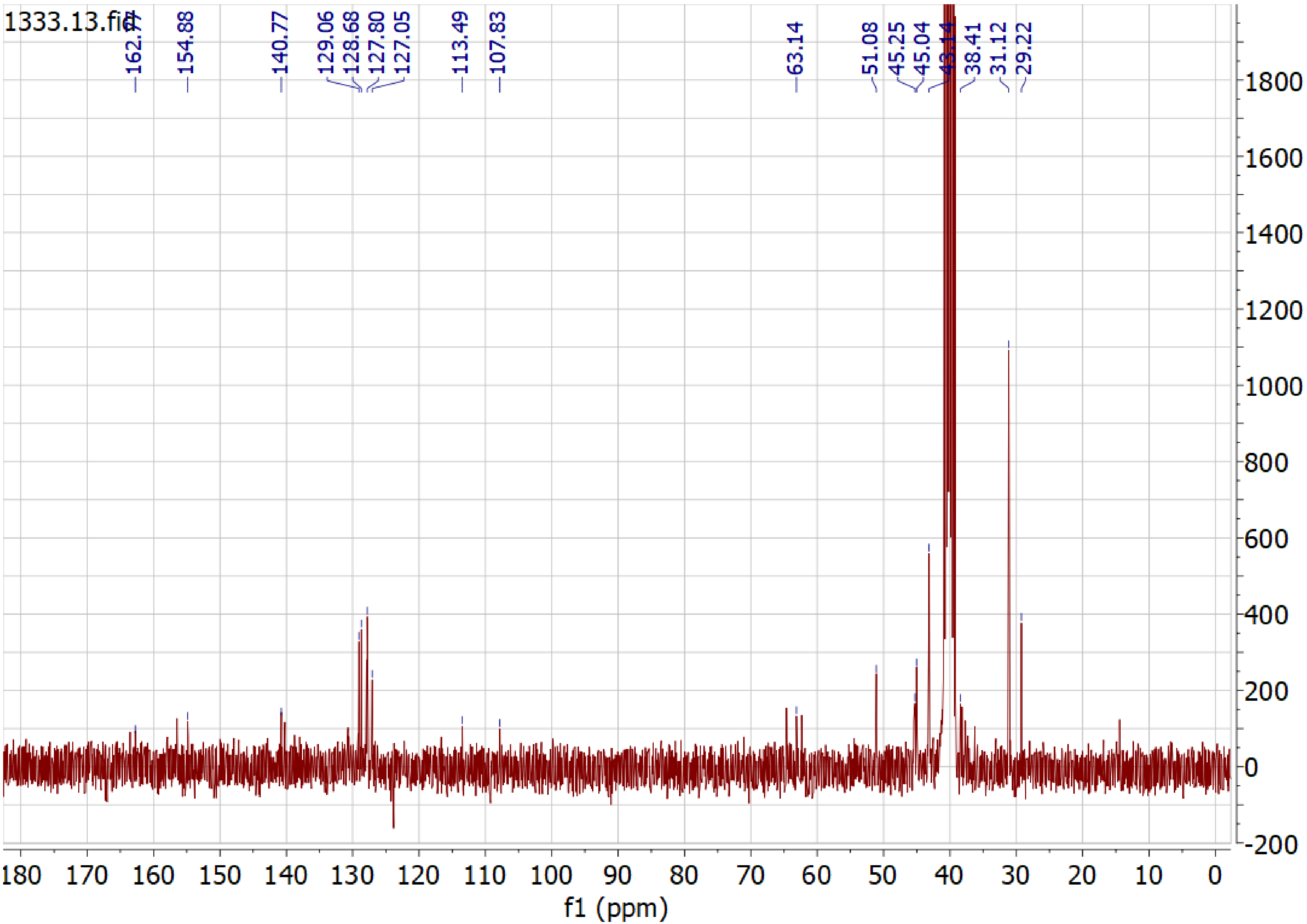

Figure S28. ^{1}H NMR of compound 8i

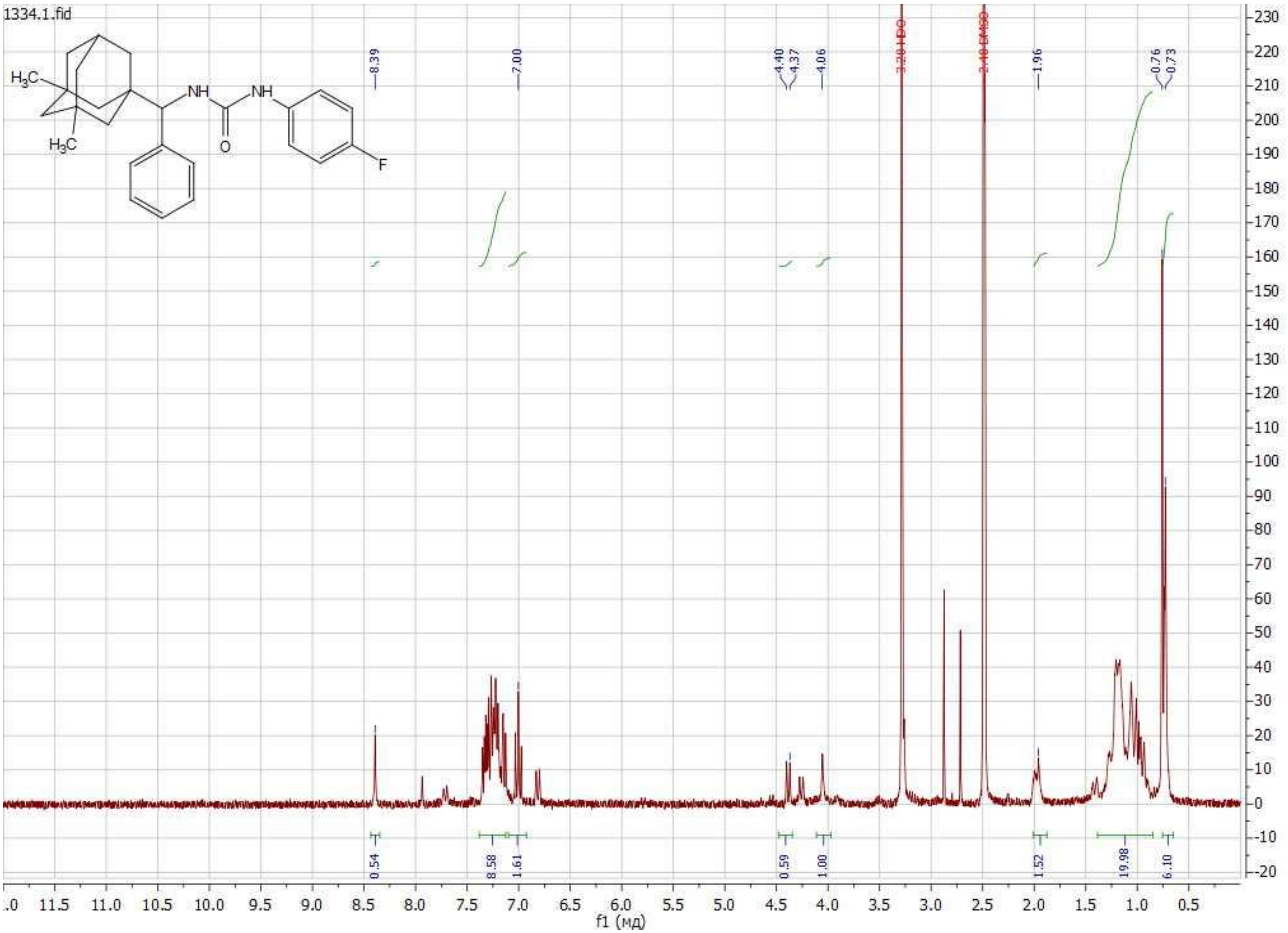

Figure S29. ^{13}**C NMR of compound 8i**

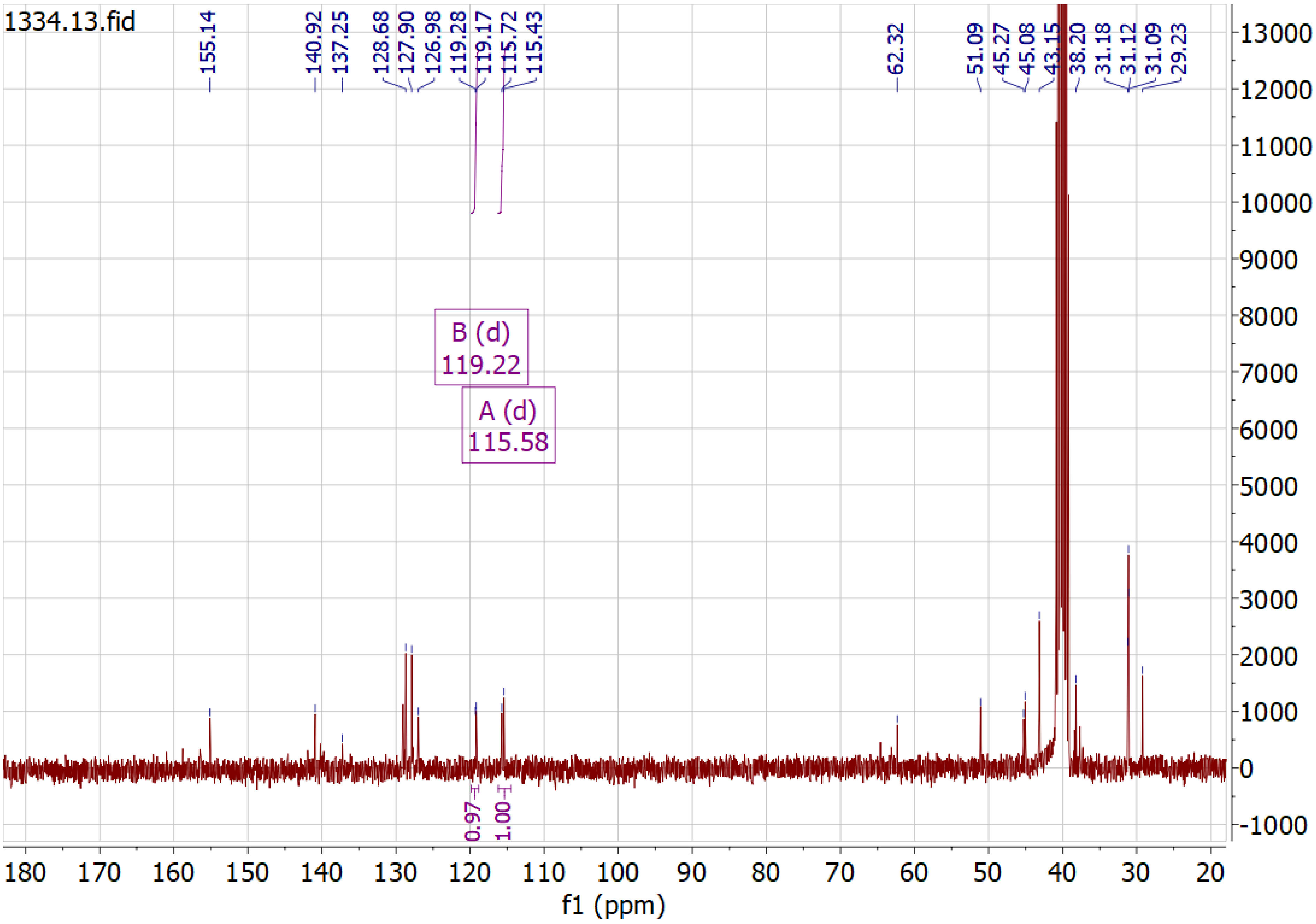

Figure S30. ^{1}H NMR of compound 8j

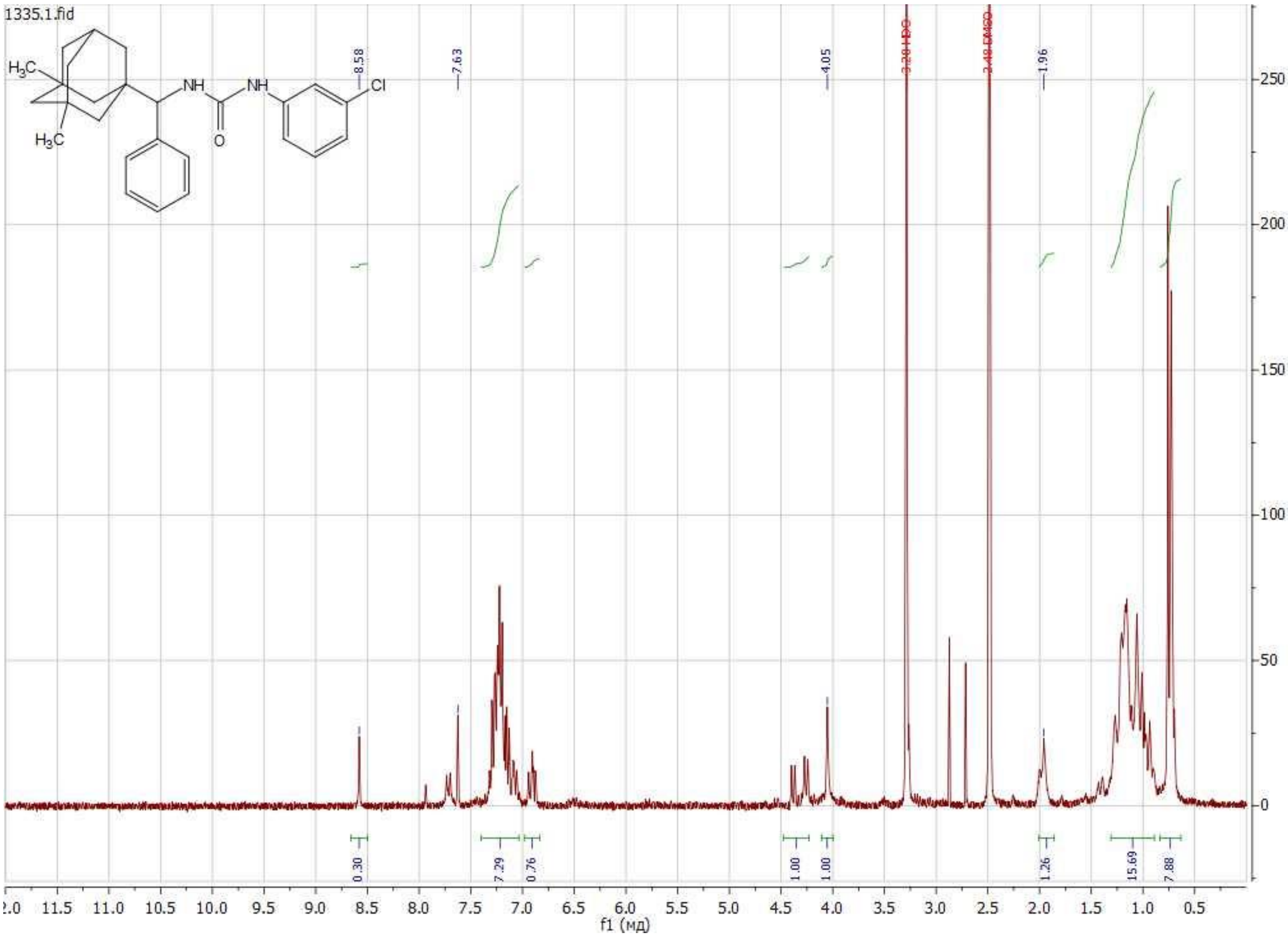

Figure S31. ^{13}C NMR of compound 8j

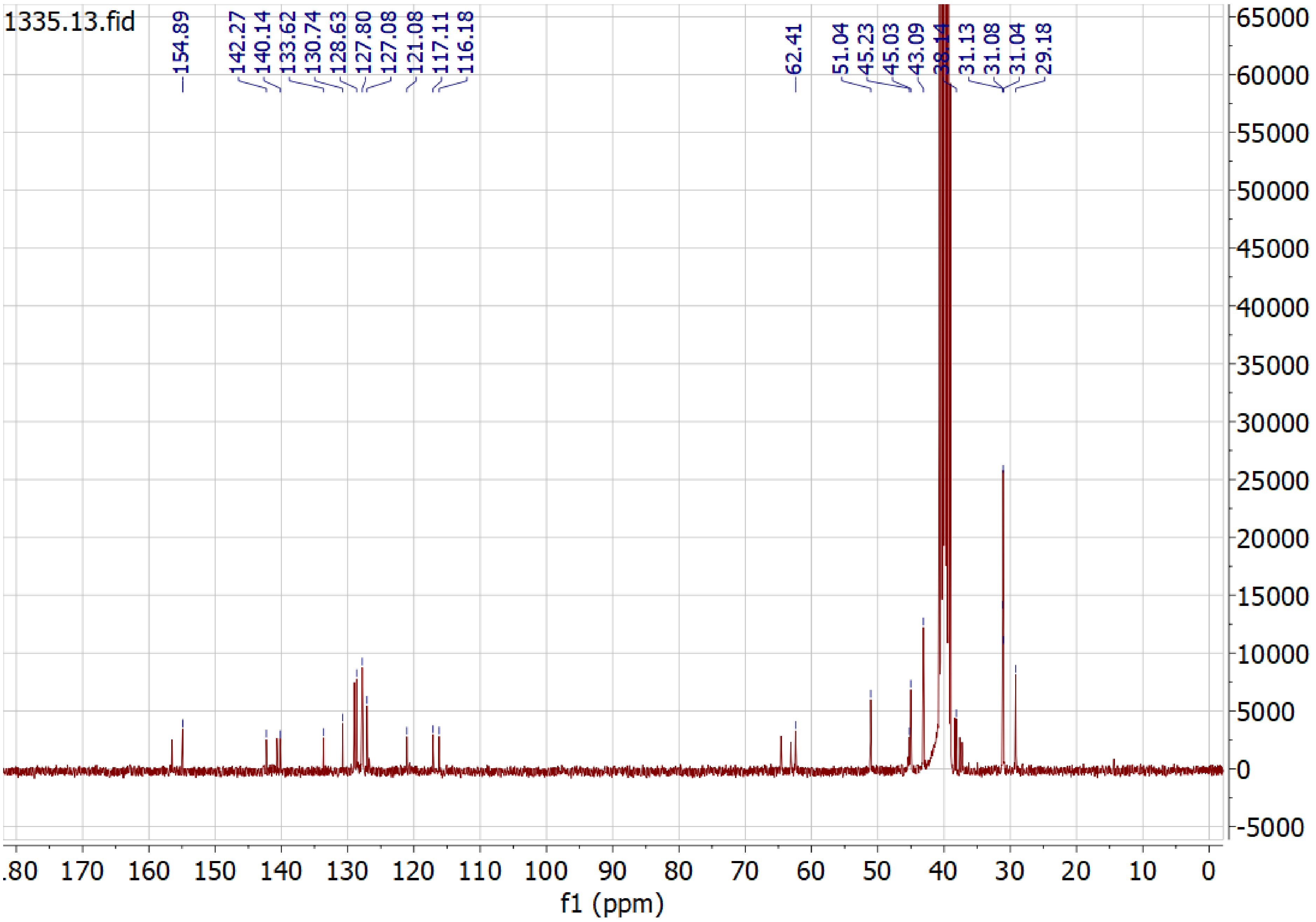

Figure S32. ^{1}H NMR of compound 8k

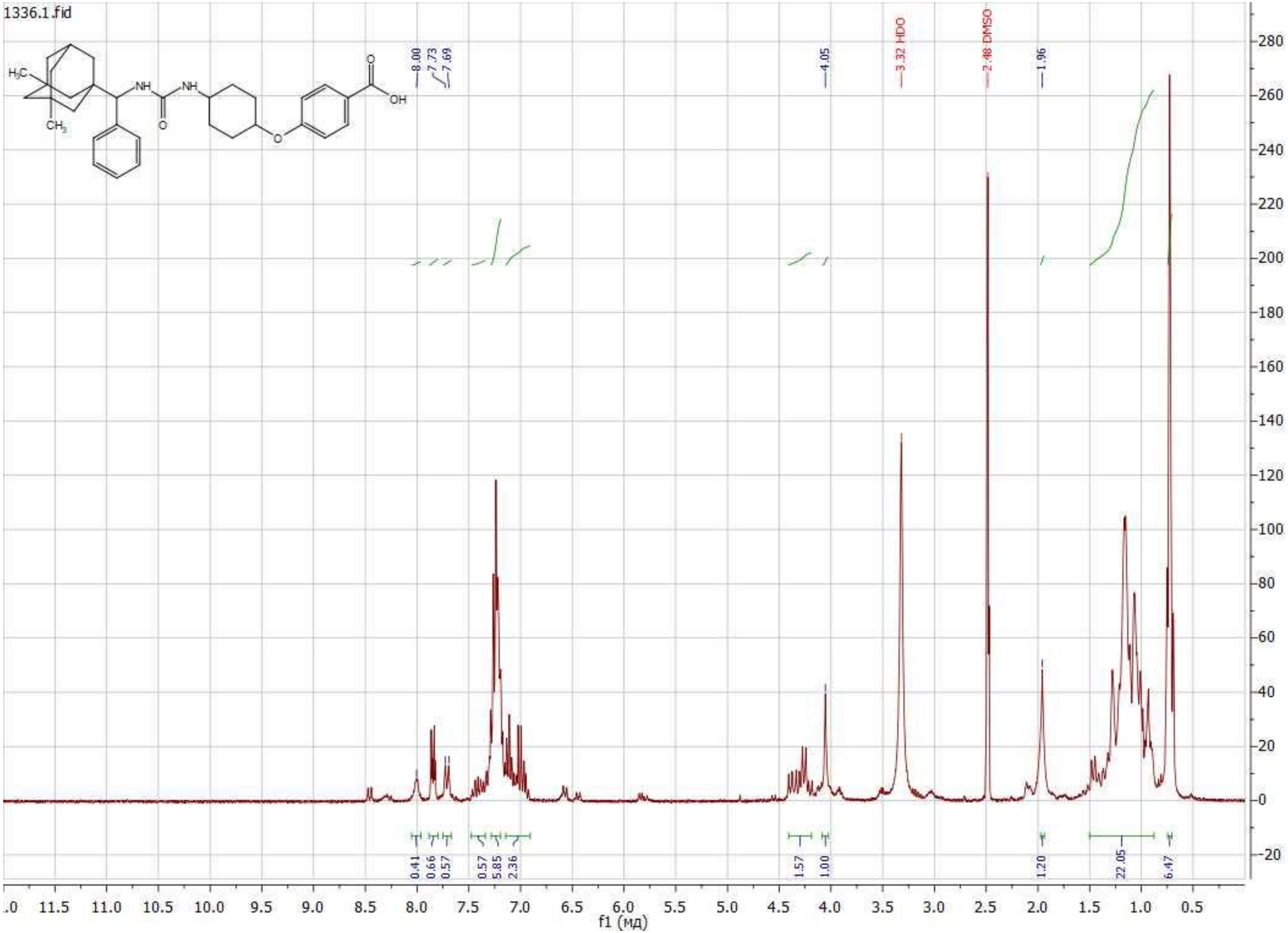

Figure S33. ^{13}C NMR of compound 8k

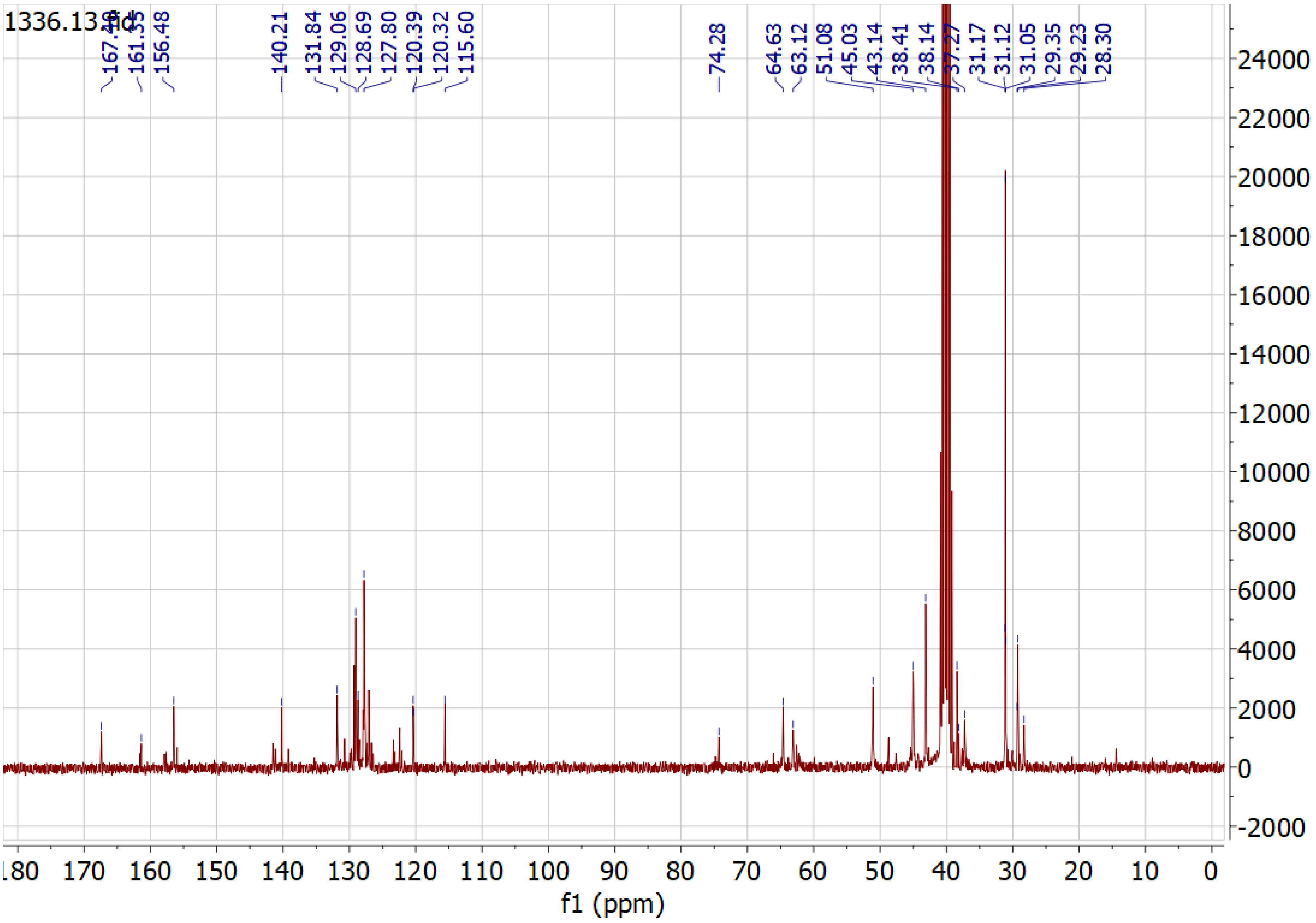

Figure S34. ^{1}H NMR of compound 10a

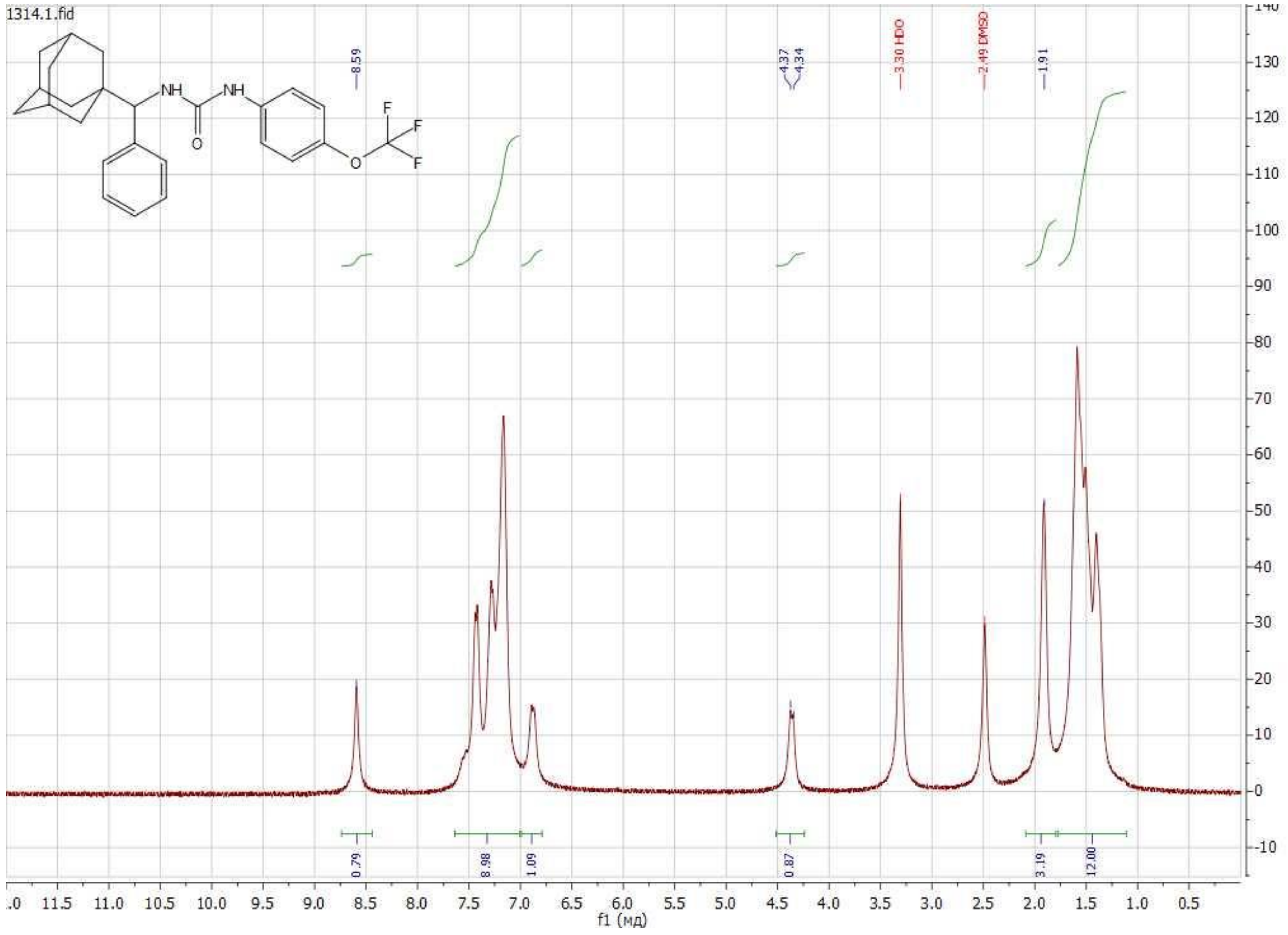

Figure S35. ^{13}C NMR of compound 10a

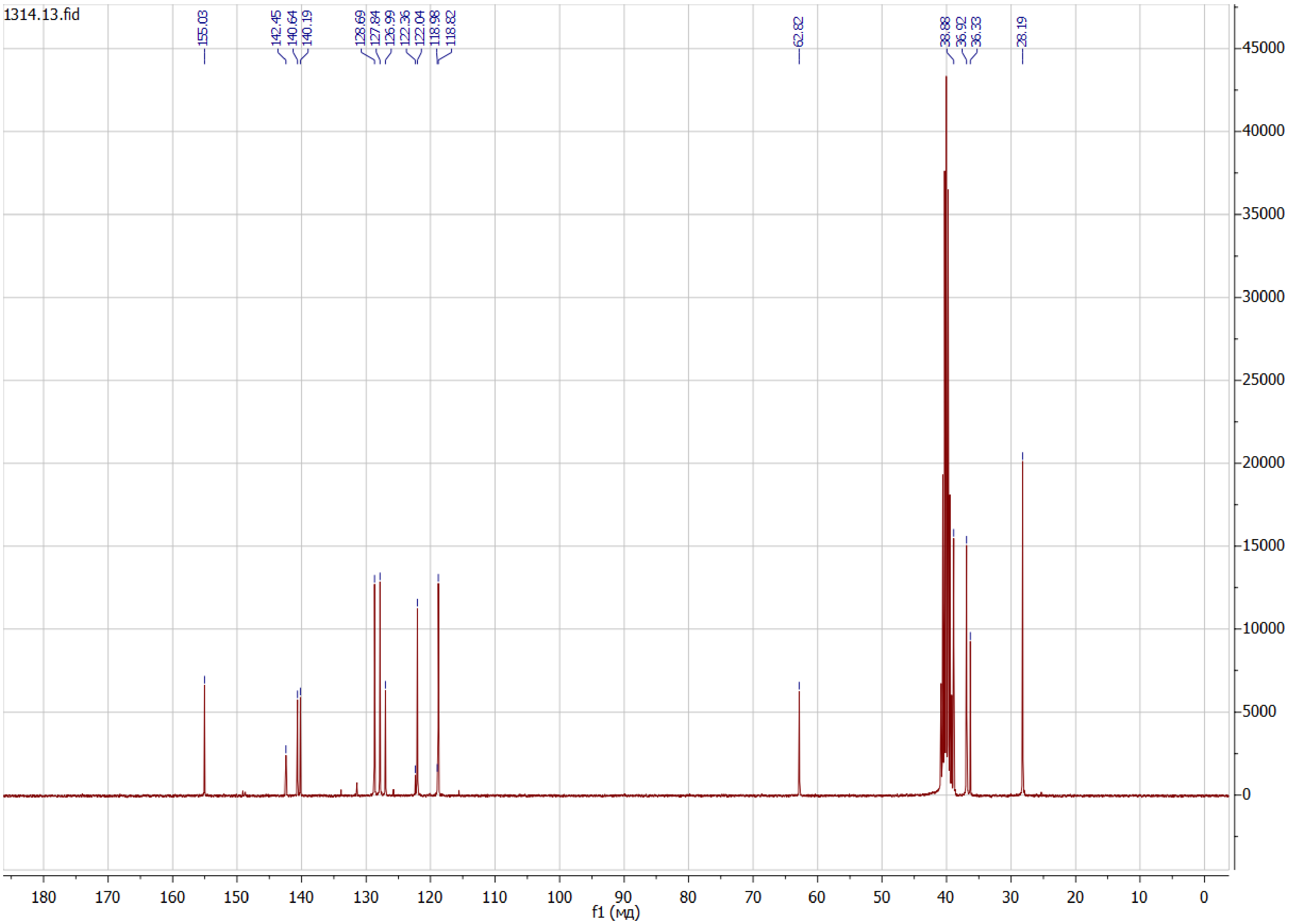

Figure S36. ^{19}**F NMR of compound 10a**

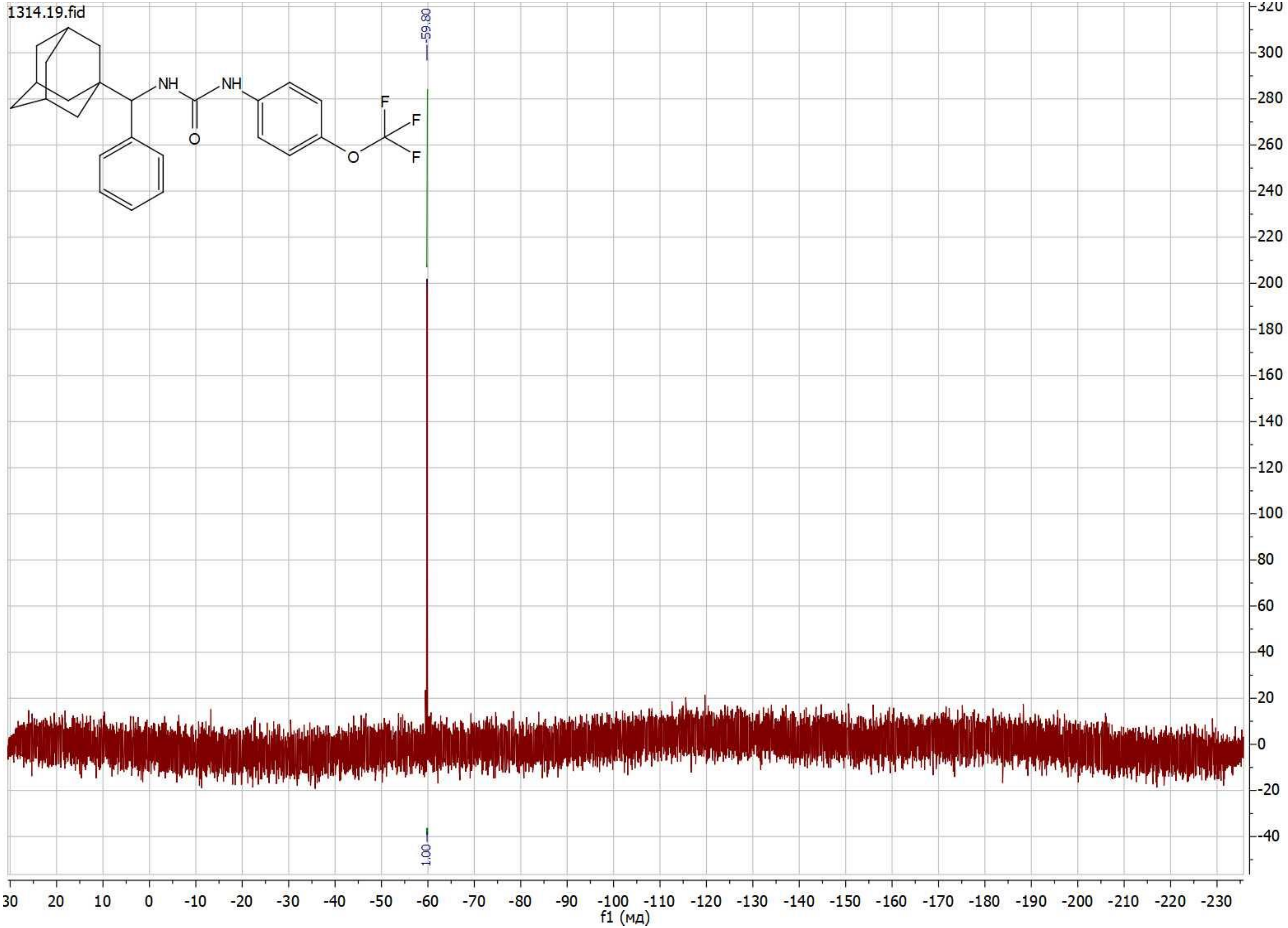

Figure S37. ^{1}H NMR of compound 10b

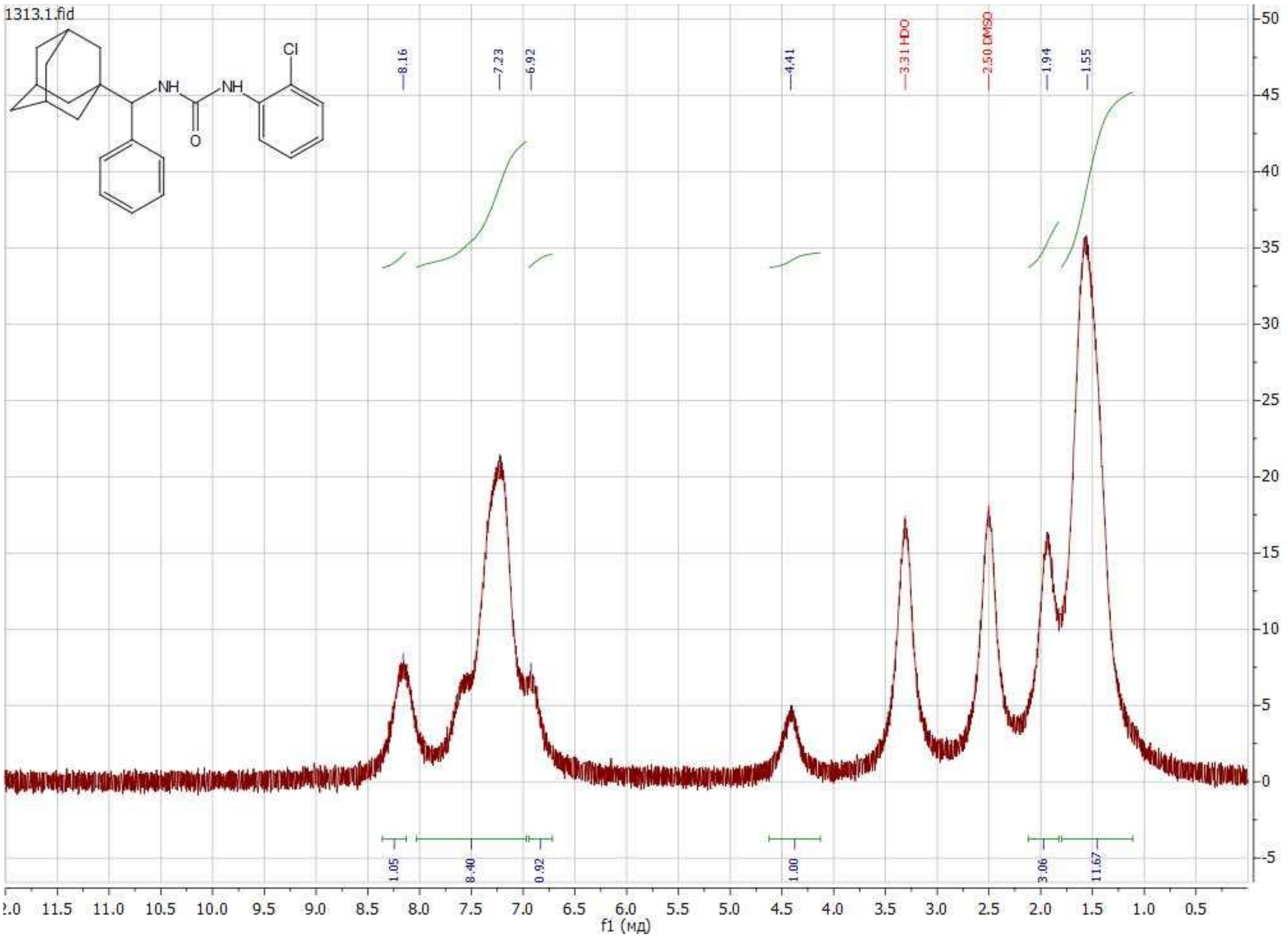

Figure S38. ^{13}C NMR of compound 10b

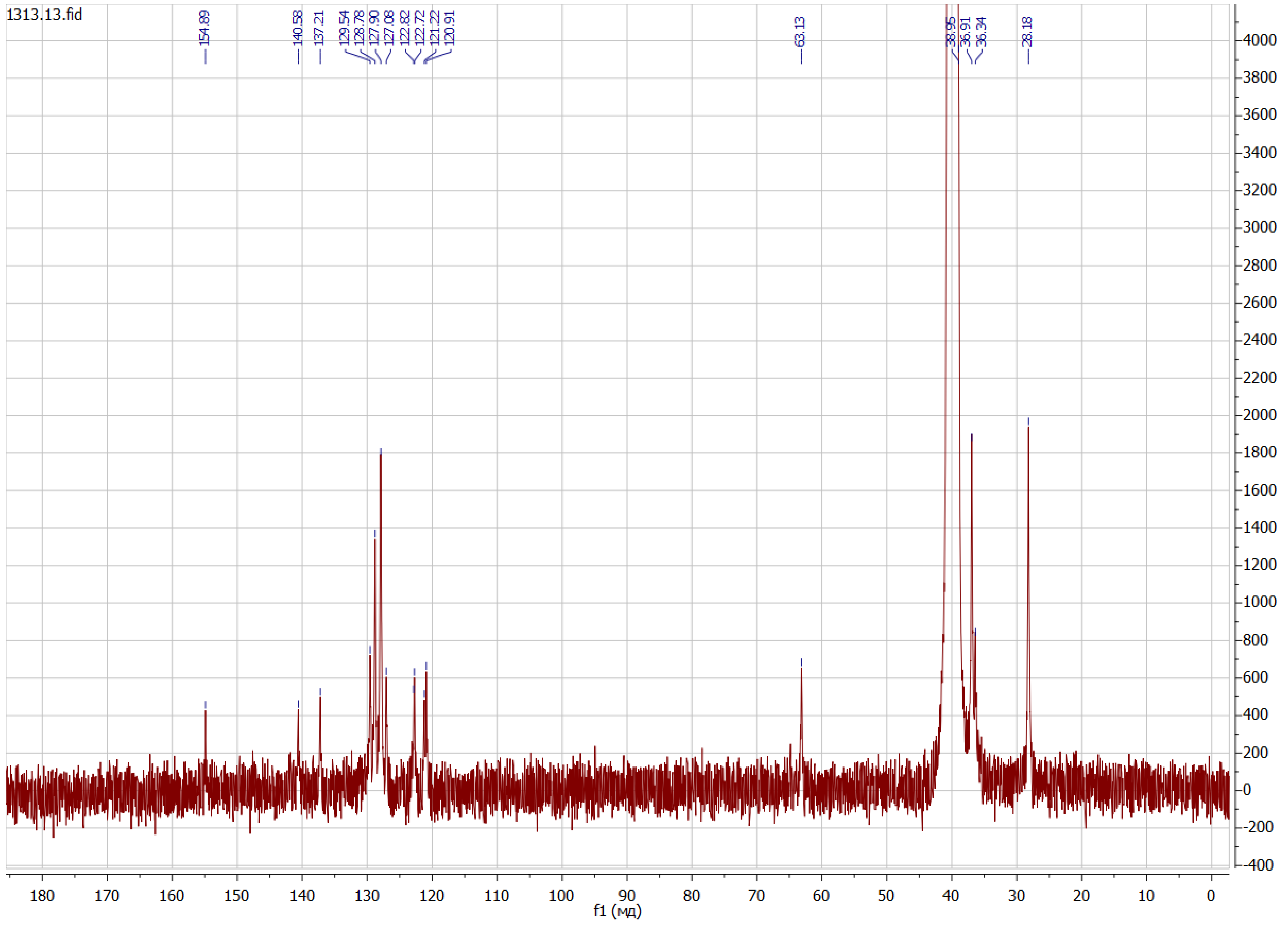

Figure S39. ^{1}H NMR of compound 10c

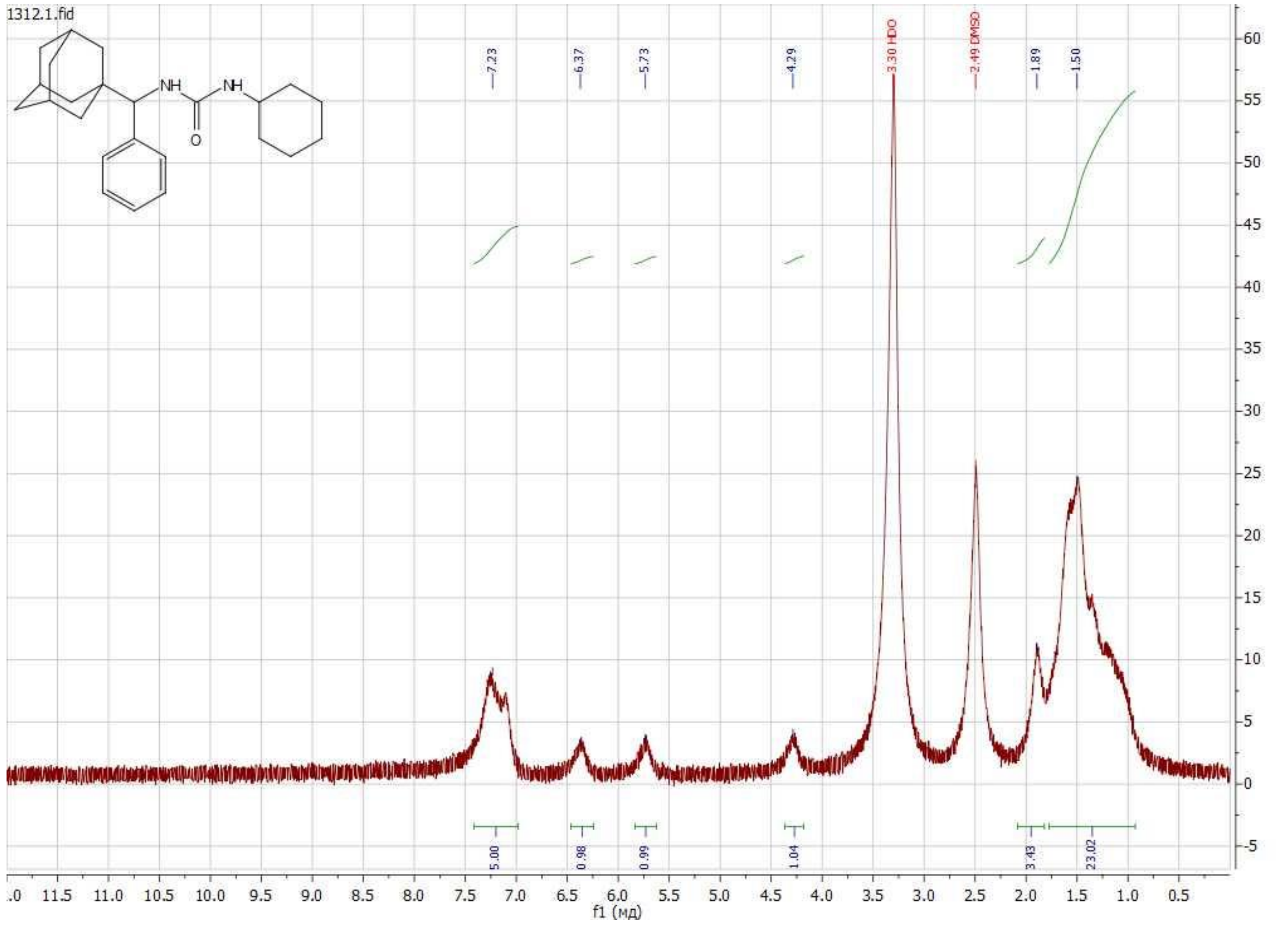

Figure S40. ^{13}C **NMR of compound 10c**

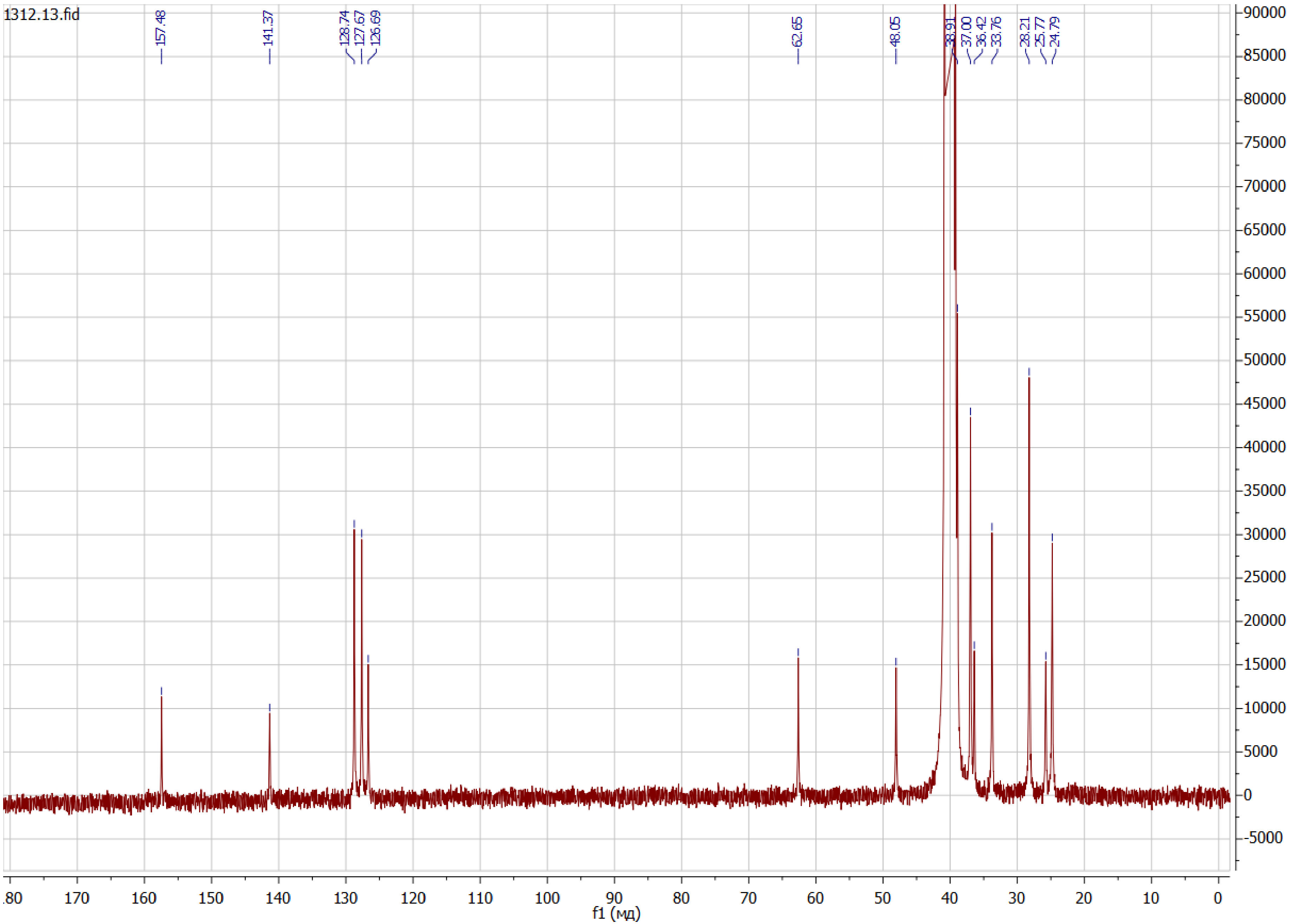

Supplement: Supplementary file 1 [file molecules-28-03577-s001.zip › molecules-2337831-supplementary.pdf]
